# *Cannabis*: from crop to shop—some insights about stability to access quality control

**DOI:** 10.1186/s42238-026-00409-9

**Published:** 2026-02-23

**Authors:** Claudete da Costa-Oliveira, João Gabriel Gouvêa-Silva, Raoul dos Santos Fernandes Muniz, Jéssica Sales Felisberto, Priscila Gava Mazzola, Renato Crespo Pereira, Davyson de Lima Moreira, Ygor Jessé Ramos

**Affiliations:** 1 School of Medical Sciences, State University of Campinas (UNICAMP), Campinas, Brazil; 2 Association to Support Medical Cannabis Research and Patients (APEPI), Campinas, Brazil; 3 Farmácia da Terra Laboratory, College of Pharmacy, Federal University of Bahia (UFBA), Salvador, Bahia Brazil; 4 School of Pharmaceutical Sciences, State University of Campinas (UNICAMP), Campinas, Brazil; 5 Department of Marine Biology, Fluminense Federal University (UFF), Niterói, Brazil; 6 Laboratory of Natural Products and Biochemistry, Rio de Janeiro Botanical Garden Research Institute (JBRJ), Rio de Janeiro, Brazil

**Keywords:** *Cannabis sativa L*, Chemical stability, Chemodiversity, Ecochemistry, Post-harvest, Essential oils, Cannabinoids

## Abstract

**Background:**

*Cannabis sativa* L. is increasingly used for medicinal and commercial purposes, yet most reviews treat chemical composition, ecology, and post-harvest processing as separate topics. This manuscript advances a conceptual framework that links chemical stability, chemodiversity, and post-harvest quality across the production chain (“crop to shop”), emphasizing and detailing how environmental and technological drivers shape cannabinoid and terpenoid profiles and, ultimately, product standardization.

**Methods:**

We conducted a structured scoping review with bibliometric mapping of studies on volatile oils from inflorescences (1963–2025), applying explicit eligibility criteria and extracting metadata on genotype, collection site, plant state, extraction procedure, and analytical platforms. To synthesize chemical patterns, we employed exploratory chemometric summaries (principal component analysis and hierarchical grouping) and comparative narrative analyses of pre*-* and post-harvest factors (*e.g.*, drying, grinding, extraction, storage, and others), and their implications for stability and quality assessment.

**Results:**

Across regions, chemical stability emerged as the central axis organizing chemodiversity, ecological roles, and quality outcomes. Pre*-*harvest conditions (cultivar, environment, phenology, and others) and post-harvest practices (drying regimes, particle size, extraction choices, storage conditions such as temperature, light, oxygen, and moisture) consistently redirected volatile and non-volatile profiles, with predictable formation or loss of key markers. The mapping highlights geographic concentration of research in Europe, the underrepresentation of the Americas, Oceania, and Africa, and limited coverage of minor cannabinoids and oxygenated terpenoids despite their technological relevance. The synthesis clarifies when technological steps favor sesquiterpenes versus monoterpenes, how trichome integrity protects active compounds, and why water activity and packaging permeability are decisive for stability. This review also identifies operational gaps regarding winterization parameters, sterilization impacts, and the scale*-*up of greener extraction methods.

**Conclusions:**

By integrating phytochemistry, chemical ecology, and stability science, this review proposes a stability–chemodiversity–quality framework that distinguishes this approach from prior reviews. The framework supports actionable guidelines for cultivation choices, post-harvest handling, extraction designs, packaging, and storage to improve reproducibility and labeling accuracy. It also sets priorities for future research: standardized stability testing across matrices, inclusion of minor constituents, ecologically informed phenology, and validation of processing models based on moisture dynamics and oxygen control.

**Supplementary Information:**

The online version contains supplementary material available at 10.1186/s42238-026-00409-9.

## Background

*Cannabis sativa* L. (Cannabaceae), a plant with a rich history spanning thousands of years, has undergone a significant transformation from a traditional crop to a source of modern medicinal and commercial products. It is a highly versatile plant known for its substantial medicinal, economic, and agricultural value (Kavousi et al. 2022). Although *C. sativa* is often classified into distinct species (*e.g.*, *C. indica* and *C. ruderalis*), the valid botanical nomenclature recognizes only a single species, *C. sativa*. The debate over species classification persists within plant taxonomy, yet recent genetic studies, free from cultural biases, support the concept of a single species encompassing multiple subspecies and varieties (McPartland and Guy 2017; McPartland and Small 2020). Nevertheless, most existing syntheses still address chemical composition, ecological drivers, and post-harvest processing as largely separate domains rather than as interacting components of a single stability-centered production system.

Despite being considered a single species, *C. sativa* has undergone extensive human manipulation over millennia. Crossbreeding, adaptation to diverse cultivation conditions, and exposure to environmental and climatic variations have led to the development of numerous varieties. Furthermore, these varieties exhibit distinct chemotypes, or chemical variations within the same species, where individual plants produce unique chemical profiles, particularly in terms of secondary metabolites (Polatoglu 2013). This chemodiversity is evident in the varying compositions of extracts derived from these plants and contributes to the broader phytochemical landscape of *Cannabis*, which encompasses a wide spectrum of cannabinoids, terpenes, and flavonoids and presents both opportunities and challenges. While chemodiversity allows the development of targeted therapies, it simultaneously complicates standardization and quality control, since the unique chemical profiles across cultivars hinder efforts to ensure product consistency, a factor particularly critical for medical *Cannabis*. Therefore, in this review, we adopt the nomenclature *C. sativa*, and consider its subspecies and varieties as distinct chemotypes, explicitly highlighting chemodiversity as a key factor influencing stability and quality along the “crop-to-shop” continuum.

The substantial chemical variability observed among *C. sativa* cultivars, and even within the same varieties grown under different environmental conditions, has been well documented (Micalizzi et al. 2021a). While several terpene synthase genes have been identified in *C. sativa*, the genetic mechanisms driving terpene variation among its cultivars remain largely unexplored. This knowledge gap presents a valuable opportunity for genomic research to enhance the accuracy and efficiency of plant identification. Additionally, such studies could significantly improve quality control and standardization efforts, ultimately supporting more effective therapies and fostering a more responsible and sustainable industry (Watts et al. 2021). To maintain the chemical stability of volatile oils (VOs), a thorough understanding of terpenoid profiles across different cultivars is essential. This knowledge can guide the selection of optimal varieties for specific cultivation processes and inform best practices for post-harvest handling. Ultimately, it contributes to the production of high-quality, chemically consistent products.

In recent years, the demand for this medicinal plant has increased markedly. However, the pressing need persists for additional scientific research and technological advancements to optimize the *C. sativa* production chain, spanning from cultivation to patient use, and encompassing operations of all scales (Bernstein et al. 2019; Gorelick and Bernstein 2017). As legal frameworks for *Cannabis* products continue to evolve, challenges associated with its cultivation, chemical composition, and quality control have become more pronounced. One of the most complex issues facing the *Cannabis* industry is the stability of its chemical constituents, particularly cannabinoids such as Δ^9^-tetrahydrocannabinol (Δ^9^-THC), cannabigerol (CBG), and cannabidiol (CBD), as well as terpenes. These compounds, being highly sensitive to environmental factors such as light, temperature, and humidity, are prone to degradation during cultivation, processing, and storage. This instability poses significant challenges to maintaining consistent product potency and quality, both of which are essential for consumer safety and regulatory compliance. Yet, despite the growing number of studies on *Cannabis* chemistry, there is still a lack of integrative frameworks that explicitly connect chemodiversity patterns with stability mechanisms and post-harvest technological choices.

Moreover, quality control measures for *Cannabis* products, spanning from cultivation to retail, are still evolving. As *Cannabis* moves through the supply chain, from cultivation and extraction to manufacturing and final sale, maintaining product integrity, preventing contamination, and ensuring accurate labeling are critical concerns. Standardized testing protocols, including those for pesticides, residual solvents, and microbial contaminants, are essential to safeguard public health and maintain consumer trust. The chemical composition of *C. sativa* is a key determinant of its applications across various industries, particularly due to its distinctive combination of cannabinoids and terpenes. While cannabinoids have been extensively studied for their medicinal properties, the role of volatile terpenes has received comparatively less attention. Recent research highlights the synergistic effects of cannabinoids and terpenes, commonly referred to as the entourage effect, which has significant implications for pharmaceutical applications (Silva Sofrás and Desimone 2022). A comprehensive understanding of the interactions and stability of these compounds throughout the production process is essential for enhancing the quality, efficacy, and sustainability of *C. sativa*-derived products. However, most previous reviews have either focused on pharmacological aspects or on analytical methods, without jointly mapping global volatilome variation, stability constraints, and post-harvest processing within a single conceptual model.

This review aims to explore key challenges and knowledge gaps by addressing essential questions, such as: (i) How can a deeper understanding of chemical stability at various stages of production contribute to improving the quality of *Cannabis*-derived products? (ii) What strategies can be developed and implemented to ensure reproducibility and optimize the cost–benefit ratio in the production of high-quality *Cannabis*?

By providing a comprehensive overview of the importance of chemical stability and variability in *Cannabis* compounds, this review considers factors such as chemical consistency, phenotypic expression, and the effects of cultivation and processing. Specifically, we propose an integrative stability–chemodiversity–quality framework that links phytochemical stability to ecophysiological and technological drivers along the *Cannabis* production chain. To achieve this, we combine a scoping review and bibliometric mapping of VOs from inflorescences with chemometric analyses of terpenoid variation and a critical appraisal of pre- and post-harvest practices. In doing so, this work goes beyond previous *Cannabis* reviews by integrating phytochemistry, ecochemistry, and stability science into a unified conceptual and operational model for quality control**.** Addressing these issues will contribute to the development of more reliable, effective, and commercially viable *Cannabis* products while promoting a more integrated and informed approach to the production chain of this medicinal plant.

## Methods

The review was conducted and reported in accordance with the PRISMA-ScR guidelines (Tricco et al. 2018), and a comprehensive scientific literature search was carried out to find studies on the characterization of volatile oils (VOs), including essential oils and volatile fractions obtained using techniques besides steam distillation and/or hydrodistillation, from *C. sativa* inflorescences, up to November 03, 2025 (date of the last search). The following sources were consulted: PubMed/MEDLINE and Google Scholar. The search strategy was formulated using Boolean operators and controlled/free terms (*e.g.*, “terpenoid*” AND “essential oil*” AND “*Cannabis*” AND “fiber hemp” AND “chemical composition”), with specific adaptations per database. There were no language restrictions; when necessary, screening was performed with assisted translation. Deduplication was performed before screening using Microsoft Excel^®^.

### Eligibility criteria

*Inclusion (*VOs *corpus):* primary, peer-reviewed studies that: (i) reported essential oils from *C. sativa* inflorescences obtained by hydrodistillation and/or steam distillation; (ii) specified the extraction method, plant part, collection site, and analytical technique (GC–MS/GC-FID/GC × GC, etc.); and (iii) presented chemical composition.

*Descriptive subdivision (other* VOs)*:* studies reporting volatile fractions obtained by SFE*-*CO₂ and volatilome studies by solid-phase microextraction (SPME) were eligible for separate narrative/cartographic synthesis, provided they met requirements (ii) and (iii) above.

*Exclusions:* reviews, commentaries, abstracts without methods, duplicates, reports without traceable analytical data, non-volatile extracts, and studies lacking clarity on the plant part or analytical method.

### Identification, screening and selection process

The dentification process occurred in two independent phases by two reviewers: screening of titles/abstracts and full-text evaluation; disagreements were resolved by consensus with a third reviewer. In total, 16,814 records were identified in the consulted sources; after deduplication, 1,850 unique records advanced to the title/abstract screening. Of these, 155 articles were evaluated in full text. Applying the eligibility criteria and distinguishing between the corpus and subcorpora, 58 studies comprised the final set for examination of *C. sativa* VOs. The PRISMA-ScR flowchart with the reasons for exclusion at each stage is shown in Figure SS1.

### Data extraction and operational definitions

Data extraction was performed in duplicate, using a pre*-*determined form, covering: country/municipality and coordinates (when available), genotype/variety/geotype/chemotype, material state (fresh/dry), extraction parameters (equipment), and analytical platform (GC–MS/GC-FID/GC × GC).

For comparability, major constituents were defined as compounds with a content > 5% in the respective profile (Supplementary Table SS1). Trace compounds and zeros were treated according to compositional data procedures. Missing coordinates were inferred at the municipal level when possible. Altitude was obtained from the website CityElevation (City elevations worldwide 2025).

### Synthesis of data and statistical/chemometric analyses

Data synthesis included: (i) a global scale (countries with eligible studies) and (ii) a regional scale focusing on Italy.

Principal Component Analysis (PCA) identified correlations among terpenoid in VOs and between terpenoids and elevation, retaining eigenvalues > 1 (Kaiser criterion). Hierarchical clustering analysis (HCA) (Unweighted Pair Group Method with Arithmetic Mean method, Euclidean distance) was used to visualize VO composition at the regional scale and global scale. A Pearson correlation matrix (α = 0.05) was generated to assess the relationship between VO composition and elevation. The correlation strength was categorized as weak (0.10–0.39), moderate (0.40–0.69), strong (0.70–1.00), and significant correlations were highlighted. Heat maps were generated with hierarchical ordering consistent with the compositional metric. Statistical significance was evaluated by Tukey’s test, and significance was set at *p* < 0.05. All analyses were performed using Statistica^®^ software version 10 (StatSoft Inc., Tulsa, OK, USA) and OriginPRO^®^ software version 10 (OriginLab, Northampton, MA, USA).

## Results and discussion

**Fig. 1:**
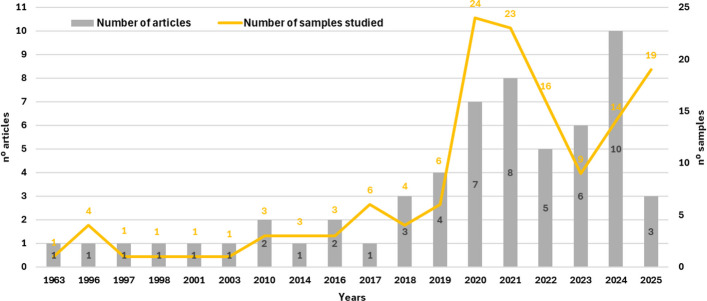
Annual number of scientific articles on volatile oils from *Cannabis* inflorescences

The bibliometric analysis identified 58 articles that characterized essential oils or volatile fractions of *C. sativa* and derivatives, encompassing 139 samples analyzed between 1963 and 2025 (Fig. 1). Until 2014, publications were sparse, with only one article in isolated years (1963, 1996, 1997, 1998, 2001, 2003, 2014) and two articles in 2010 and 2016, indicating that systematic characterization of the volatiles of *Cannabis* remained marginal in the phytochemical literature for roughly half a century. From 2018 onwards, however, a clear inflection point is observed, with a progressive increase in both the number of articles and samples, reaching 5–10 publications per year between 2020 and 2024. The period 2018–2024 accounts for the bulk of the output, with 43 of the 58 articles and approximately 70% of the samples, and a peak of 10 publications in 2024. These findings indicate a recent and accelerated consolidation of the field, consistent with regulatory changes and the growing interest in *Cannabis*-based products for medicinal and industrial applications.

This recent expansion is grounded in a longer historical trajectory of research on the volatile fraction of *Cannabis* that, although not included in the present dataset because these studies did not meet the predefined inclusion and exclusion criteria, established the conceptual and analytical foundations of the field. The earliest of these is the classic study by Simonsen and Todd (1942) (Simonsen and Todd 1942), conducted in Alexander Robertus Todd’s group at the University of Manchester, which distilled and fractionated the essential oil of Egyptian hashish (Africa) and identified *p*-cymene, *p*-cymenene (1-methyl-4-isopropenylbenzene) and the sesquiterpene humulene (α-caryophyllene), thereby inaugurating the nominal identification of terpenes in *Cannabis* matrices. Two decades later, Martin et al. (1961) (Martin et al. 1961), in a collaboration between the Commonwealth Laboratories in Melbourne and the Food and Drug Directorate in Ottawa, applied gas chromatography to the essential oils from fresh *C. sativa* leaves, describing β-myrcene, limonene, α-humulene, and β-caryophyllene as major constituents and proposing the volatile profile as a tool for botanical and forensic identification. Subsequently, Nigam et al. (1965) (Nigam et al. 1965), working with authentic Indian *C. sativa* material analyzed at the Regional Research Laboratory in Jammu Tawi in partnership with the Food and Drug Directorate, presented the first systematic characterization of the essential oil of “marihuana” under reduced pressure, revealing a broad set of mono- and sesquiterpenes, among which caryophyllene (45.7%) and humulene (16.0%) were predominant. Together, these three methodological milestones established the framework underpinning the contemporary intensification of research on *Cannabis* essential oils, as reflected in the rise in publications observed since 2018 (Simonsen and Todd 1942; Martin et al. 1961; Nigam et al. 1965; Mechoulam and Hanuš 2000).

### Geographic variation in the chemical diversity of *Cannabis* cultivars

A comparison of scientific production on a latitudinal scale revealed a significant geographic disparity in research focus. Most studies have been conducted on species and environments in Europe, particularly in Italy (41.8% of studies), compared to the Americas. This discrepancy can be attributed to the greater concentration of *C. sativa* related to research efforts and resources in Europe, likely driven by more established regulatory frameworks, funding opportunities, and academic expertise in the region. Conversely, research in North and South America has been relatively limited, likely reflecting differences in legislative restrictions, historical stigmas, and the slower integration of *Cannabis* research into academic and industrial domains.

#### Global scale

**Fig. 2:**
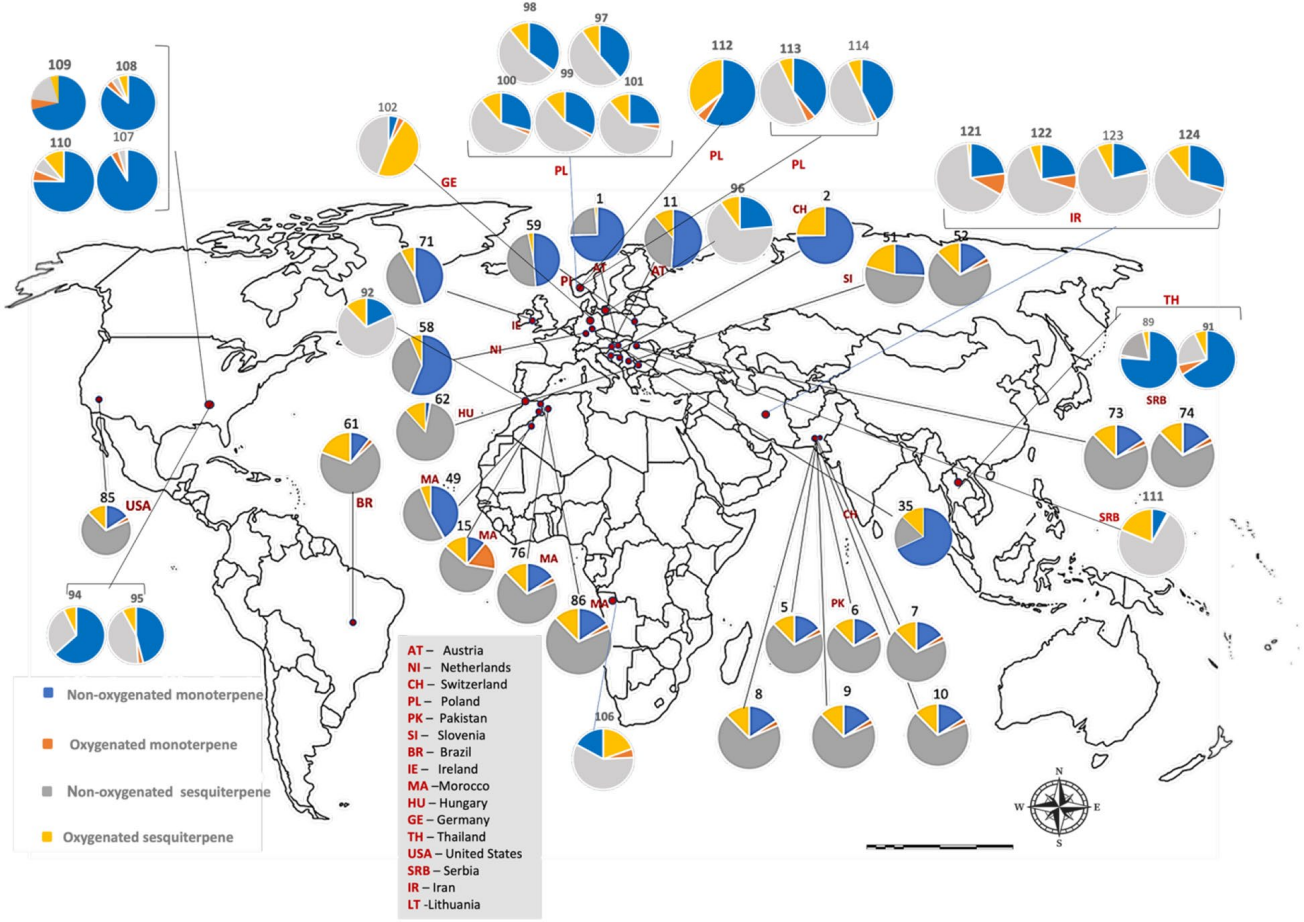
Global variability of terpenoid classes in *C. sativa* with a terpene-based chemotype. Pie charts represent the mean relative contribution of non-oxygenated monoterpenes, oxygenated monoterpenes, non-o xygenated sesquiterpenes and oxygenated sesquiterpenes (compounds ≥ 5%) for each locality with available VO data, based on collection sites reported in the original studies (see Supplementary Table SS1 and SS2). Source: Map generated by the authors using Microsoft PowerPoint

*C. sativa* is currently cultivated and/or authorized for medicinal or industrial use in many countries worldwide. In Europe, approximately 28 countries have already regulated its medical use, and some of them also allow controlled cultivation, whereas in the Americas and Asia only a limited number of countries have adopted comparable regulations (Riaz and Akram 2024). However, the geographical coverage of VO studies is much narrower than the regulatory landscape. The compiled dataset comprises more than one hundred VO profiles from cultivars or naturalized populations in 16 countries distributed across Europe (Austria, the Netherlands, Switzerland, Italy, Poland, Slovenia, Serbia, Hungary, Germany, Ireland, and Lithuania), North Africa (Morocco), Asia (Pakistan, Iran, and Thailand) and the Americas (Brazil, and the USA (United State of America)), with Italy treated separately owing to the disproportionately large number of Italian samples (Fig. 2; Supplementary Table SS1 and Table SS2). These records span almost three decades of research and encompass distinct genetic backgrounds, agronomic systems, and post-harvest conditions.

The global distribution of the four major classes of terpenoids is summarized in Fig. 2. For each locality, VOs were classified according to the cumulative relative abundance of non-oxygenated monoterpenes, oxygenated monoterpenes, non-oxygenated sesquiterpenes, and oxygenated sesquiterpenes, considering only constituents present at ≥ 5% in at least one sample (Supplementary Table SS1). Across almost all regions, non-oxygenated monoterpenes (blue) and non-oxygenated sesquiterpenes (grey) occupy the largest sectors of the pie charts, whereas oxygenated terpenoids (orange and yellow) generally form smaller but chemically informative fractions of the volatile profile. Non-oxygenated sesquiterpenes, particularly β-caryophyllene and α-humulene, are consistently detected on all continents, usually accounting for 20–50% of the VO fraction and occurring in fairly constant ratios (β-caryophyllene typically two- to four-fold higher than α-humulene). In contrast, high proportions of oxygenated monoterpenes are geographically restricted and are mainly associated with chemovars from Morocco, Pakistan, and Brazil, with only minor contributions in most European and North American profiles. Oxygenated sesquiterpenes showed the opposite trend: they are negligible or present at trace levels in several central and Northern European and North American samples but may constitute more than one third of the total VOs in specific chemovars from Italy, Poland, Morocco, and Brazil. Together, these patterns reveal a strong Euro-Mediterranean bias in the available VO data and indicate region-specific trends in oxidative terpenoid metabolism in *C. sativa*.

A joint analysis of VO profiles from *C. sativa* inflorescences compiled from the literature revealed that the global chemical variability is structured by a relatively small set of mono- and sesquiterpenes (Supplementary Table SS1). Among the non-oxygenated monoterpenes, β-pinene, limonene, *trans-*β-ocimene, myrcene, and α-pinene stand out as recurrent and often predominant constituents (Ross and ElSohly 1996; Bertoli et al. 2010; Da Porto et al. 2014; Naz et al. 2017). Within the oxygenated monoterpene fraction, eucalyptol (1,8-cineole), linalool, estragole, carvone, and linalyl acetate emerge as key components in several aromatic chemotypes (Naz et al. 2017; Górski et al. 2016).

The sesquiterpene portion is dominated by hydrocarbons such as *E-*β-caryophyllene, *trans-*α-bergamotene, γ-elemene, *cis-*β-farnesene, β-selinene, δ-cadinene, and selina-3,7(11)-diene (Da Porto et al. 2014; Zengin et al. 2018; Fiorini et al. 2019; Judžentienė et al. 2023). Oxygenated sesquiterpenes—nerolidol, humulene epoxide II, guaiol, isoaromadendrene epoxide, β-eudesmol, cubenol, 10-*epi*-γ-eudesmol, oxidized derivatives of caryophyllene, and α-bisabolol—generally occur at lower levels, but become dominant in a subset of accessions, particularly those from Rovigo (Italy) (Pieracci et al. 2021; Smeriglio et al. 2020), Brazil (Soares et al. 2023), and Germany (Luca et al. 2024). Despite their lower relative abundance in most VOs, these oxygenated mono- and sesquiterpenes exhibit well-documented antibacterial, antifungal, and anti-inflammatory activities, conferring considerable technological potential for the valorization of *Cannabis* supply-chain by-products in the agri-food, cosmetic, nutraceutical, and pharmaceutical sectors (see references in the present article).

The composition matrix comprising 35 volatile constituents for all compiled samples is presented in Supplementary Table SS1, organized by city, country, and reference. The individual scores of the first two principal components (PC1 and PC2) for each VO are provided in Supplementary Figure SS2, whereas the loading coefficients of the 35 terpenoids on the PCs are listed in Supplementary Table SS3.

**Fig. 3:**
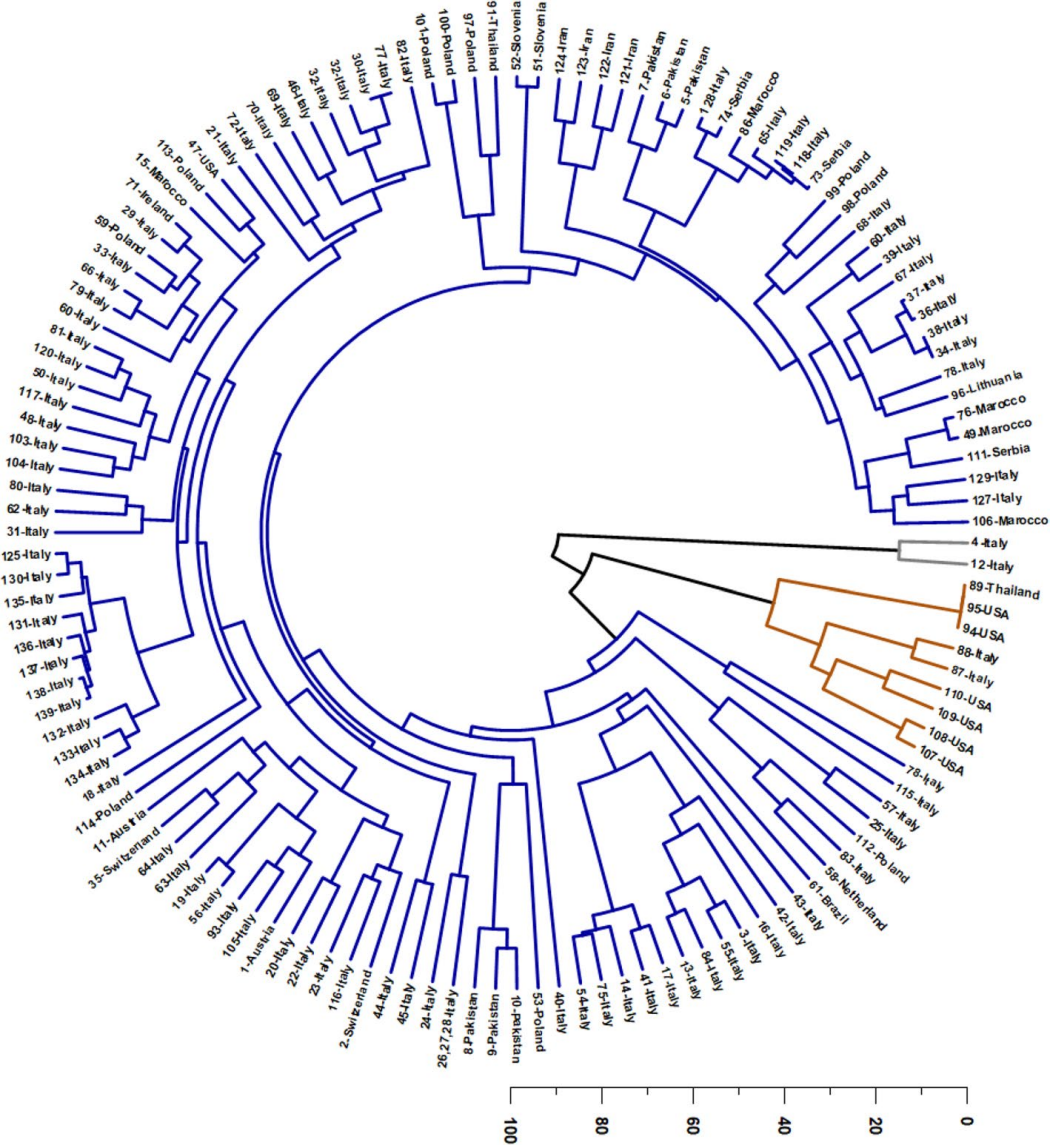
Hierarchical Cluster Analysis (HCA) of *Cannabis* volatile-oil samples based on the relative abundance of 35 major terpenoids (≥ 5% in at least one sample). The circular dendrogram resolves three well-separated chemotypic clusters (color-coded): (i) an oxidized sesquiterpene profile dominated by caryophyllene oxide and caryophylla-4(14),8(15)-dien-5-ol (grey); (ii) a monoterpene-rich profile characterized by very high myrcene (orange); and (iii) a major sesquiterpene-hydrocarbon profile dominated by *E*-β-caryophyllene and α-humulene, with variable contributions of α-pinene, myrcene, and minor oxidized sesquiterpenes, forming structured subclusters along gradients of monoterpene enrichment and sesquiterpene oxidation (blue). Sample identities and detailed compositions are provided in Supplementary Table SS1

The HCA, performed on the relative abundances of the selected mono- and sesquiterpenes, organized the VOs into three major clusters, with two small sets of atypical profiles forming isolated terminal tips in the circular dendrogram (Fig. 3). This structure indicates that, although the dataset contains a dominant “basal” chemical background shared by most accessions, a restricted number of samples diverge sharply toward extreme compositions. In practical terms, the dendrogram highlights a continuum governed by three coupled dimensions: (i) the relative dominance of hydrocarbon monoterpenes (increasing chemical “lightness” and volatility), (ii) the structural contribution of sesquiterpene hydrocarbons—particularly *E*-β-caryophyllene and α-humulene—defining the canonical *Cannabis* volatile scaffold, and (iii) the degree of sesquiterpene oxidation, which may reflect intrinsic metabolic predisposition and/or chemical evolution during drying, storage, and processing. Therefore, rather than representing fully discrete chemotypes only, the HCA delineates a chemical landscape in which a large and cohesive sesquiterpene-centered domain is flanked by two chemically extreme and compositionally simplified domains.

The largest and most representative cluster (Cluster 3) corresponds to the classical sesquiterpene-based chemotype, dominated by *E*-β-caryophyllene and α-humulene, while retaining moderate but persistent contributions of myrcene and α-pinene. This cluster encompasses most European industrial hemp samples, primarily from Italy (Abruzzo, Fiuminata, Amandola, Ascoli Piceno, Friuli-Venezia, Tuscany), but also from Austria, Slovenia, Lithuania, Switzerland, and Poland, as well as accessions from Ireland, Morocco, Pakistan, Iran, and Brazil (Da Porto et al. 2014; Zengin et al. 2018; Fiorini et al. 2019; Judžentienė et al. 2023; Soares et al. 2023; Benelli et al. 2018; Vuerich et al. 2019; Mazzara et al. 2022; Zheljazkov et al. 2020; Nafis et al. 2019; Kabdy et al. 2024).

In the updated dataset (Supplementary Table SS3), the basal signature of this major domain is exemplified by contributions of *E*-β-caryophyllene, α-humulene, myrcene, and α-pinene, together with secondary levels of caryophyllene oxide and terpinolene. This profile defines a chemically coherent “core” in which sesquiterpenes provide the dominant mass contribution, but monoterpenes remain non-negligible and may modulate organoleptic properties and functional potential.

Importantly, Cluster 3 does not fragment into sharply separated internal branches; instead, it displays gradual intracluster gradients, consistent with small-to-moderate shifts in the monoterpene fraction (*e.g.,* myrcene/α-pinene/terpinolene variability) and in the intensity of sesquiterpene oxidation, rather than the existence of additional stable major chemotypes. Within this broad group, some samples show a more sesquiterpene-rich tendency, particularly materials from Mediterranean or adjacent regions (*e.g.,* Morocco and Serbia in the compiled dataset), which occasionally coincide with increased caryophyllene oxide, suggesting a progressive drift along a sesquiterpene/oxidation axis rather than the emergence of an independent, fourth cluster.

In chemical terms, Cluster 3 is best interpreted as the baseline sesquiterpene scaffold of *Cannabis* VOs, in which the *E*-β-caryophyllene/α-humulene pair operates as a robust chemotaxonomic signature, while monoterpenes contribute to variability in “top-note” composition and aroma brightness. This balance is compatible with the recurrent dominance of *E*-β-caryophyllene observed in European hemp accessions, frequently accompanied by α-humulene at lower but consistent levels, and modulated by myrcene and α-pinene at intermediate ranges (Da Porto et al. 2014; Zengin et al. 2018; Fiorini et al. 2019; Judžentienė et al. 2023). In addition, the presence of oxygenated sesquiterpenes at low-to-moderate levels (*e.g., *caryophyllene oxide) suggests that oxidative transformations are not exclusive to outliers, but rather represent a secondary layer of variation superimposed on the classical sesquiterpene base.

A second, clearly separated monoterpene-rich cluster (Cluster 2) is defined by a strongly myrcene-dominant pattern, with limonene as the major co-constituent and only residual levels of the sesquiterpene hydrocarbons that typify Cluster 3. This cluster includes 89-Thailand, Italian samples 87–88, and North American cultivars (94–95-USA and 107–110-USA). According to Supplementary Figure SS2 and Supplementary Table SS3, this chemotype is characterized by high myrcene levels, and a markedly reduced (*E*)-β-caryophyllene, indicating a pronounced collapse of the canonical sesquiterpene scaffold into a profile dominated almost exclusively by hydrocarbon monoterpenes. This compositional pattern supports its separation as a “myrcene/limonene axis”, widely described for aromatic medicinal and recreational cultivars (Ross and ElSohly 1996; Noriega-Rivera et al. 2023; Chaiwangrach et al. 2025a; Ovidi et al. 2022; Šovljanski et al. 2024; Juliano et al. 2024; Novak and Franz 2003). The multivariate isolation of Cluster 2 is chemically coherent: the combination of extremely high myrcene and elevated limonene enhances volatility and monoterpene-driven aroma expression, while the near-depletion of *E*-β-caryophyllene and α-humulene sharply differentiates these oils from the industrial hemp baseline (Agnieszka et al., 2016; 2020; Rejdali et al., 2024). Consequently, Cluster 2 can be interpreted as the monoterpene extreme of the overall continuum, representing a distinct functional and sensory class compared with sesquiterpene-structured VOs. In applied terms, this cluster is relevant because it concentrates traits associated with monoterpene “lightness” and “brightness”, features often linked to cultivar selection in fragrance-driven and organoleptic applications.

A third cluster (Cluster 1) is represented by an oxidation-dominated and highly atypical profile comprising only two Italian samples (4-Italy and 12-Italy), which appear as isolated terminal tips in the dendrogram. Chemically, this group is defined by an almost binary composition dominated by caryophyllene oxide and caryophylla-4(14),8(15)-dien-5-ol, with the virtual absence (or residual traces) of the major monoterpenes and sesquiterpene hydrocarbons present in the remaining dataset. This unique signature indicates an extreme enrichment in oxygenated sesquiterpenes and suggests intense chemical transformation. Such a profile is compatible with two non-mutually exclusive explanations: (a) a strong intrinsic metabolic bias toward oxygenated derivatives, and/or (b) post-harvest oxidation phenomena that convert *E*-β-caryophyllene and related precursors into oxygenated products, thereby simplifying the mixture and amplifying specific oxidation markers. Notably, the predominance of caryophyllene oxide, coupled with a complementary oxygenated sesquiterpene alcohol, indicates a chemical “state” distinct from the typical baseline oils, and supports the interpretation of Cluster 1 as an oxidation extreme within the dataset.

Taken together, the HCA patterns support three coherent multivariate drivers that can be conceptually framed as orthogonal chemical axes: (1) a volatility/lightness axis governed by hydrocarbon monoterpenes such as myrcene, α-pinene, and terpinolene, which pushes Cluster 2 away from the sesquiterpene-structured background; (2) a sesquiterpene structural axis driven by *E*-β-caryophyllene and α-humulene, which maintains Cluster 3 as a compact and dominant domain; and (3) an oxygenation axis, which isolates Cluster 1 through a strong enrichment in caryophyllene oxide and related oxygenated sesquiterpenes. This tri-axial interpretation reconciles the apparent coexistence of (a) a large chemically coherent cluster, (b) a monoterpene-dominant extreme, and (c) an oxidation-dominant extreme, and explains why the dendrogram shows long isolated branches despite overall continuity in the majority of samples.

The PCA applied to the matrix of volatile constituents indicated that PC1 and PC2 explain 58.01% and 16.45% of the total variance, respectively (Supplementary Figure SS2). Consistent with the HCA, the PC1 × PC2 plane provides a structured map of the chemical space, revealing that most samples occupy a relatively compact region while the atypical profiles separate along chemically interpretable directions. PC1 captures the principal dispersion of the dataset and reflects the balance between sesquiterpene-structured oils and profiles shifted towards alternative terpene mixtures, whereas PC2 provides a secondary discriminatory axis that differentiates myrcene-enriched monoterpene profiles from oils enriched in oxygenated sesquiterpenes, including caryophyllene oxide and caryophylla-4(14),8(15)-dien-5-ol (Supplementary Figure SS2). In this multivariate context, the PCA does not merely confirm clustering; it clarifies that Cluster 2 and Cluster 1 represent chemically simplified extremes aligned with monoterpene dominance and oxygenation dominance, respectively.

Scores ranged from approximately –0.98 to + 0.11 on PC1 and –0.51 to + 0.87 on PC2 (Supplementary Table SS3). The myrcene-dominant samples belonging to Cluster 2 (e.g., 87–88-Italy, 89-Thailand, and the USA set) occupy the upper portion of the score plot, consistent with their monoterpene-rich and sesquiterpene-depleted signature.

In contrast, the two oxidation-dominated Italian samples (4-Italy and 12-Italy) are displaced towards negative PC2 values, consistent with the separation expected for oils dominated by oxygenated sesquiterpenes. The remaining samples, assigned to the basal Cluster 3, occupy the central-to-lower regions, forming a continuous distribution that mirrors moderate internal shifts in monoterpene abundance and oxidative enrichment while preserving the dominant *E*-β-caryophyllene/α-humulene backbone.

The correspondence between PCA and HCA is therefore robust: Cluster 2 aligns with the myrcene/limonene pole, Cluster 1 aligns with the oxidation-enriched extreme, and Cluster 3 defines the classical sesquiterpene baseline. This integrated interpretation supports the view that VOs composition is shaped by a complex interaction among genotype, geographic origin, cultivation context, and post-harvest handling.

In particular, the dominance of Cluster 3 across multiple countries suggests that the sesquiterpene-centered scaffold is widespread and stable across industrial hemp materials, whereas the emergence of Cluster 2 and Cluster 1 reflects targeted cultivar selection and/or chemical evolution processes that shift oils toward monoterpene intensity or oxidative dominance. From an applied perspective, this multivariate organization provides a rational framework for selecting germplasm and processing strategies: monoterpene-rich oils (Cluster 2) may be prioritized for aroma-forward applications, while sesquiterpene-structured profiles (Cluster 3) represent the most prevalent industrial baseline, and oxidation-dominated profiles (Cluster 1), although rare, may constitute a concentrated source of oxygenated sesquiterpenes relevant for specialized bioactivity-oriented or high-value-added niche applications.

Overall, this pattern suggests a complex interaction among genotype, geographic origin, and cultivation management and post-harvest processing practices in determining VO composition. As widely demonstrated for other aromatic species, essential oil composition reflects the interplay between genetic architecture and biotic and abiotic factors—climate, soil type, altitude, and interactions with microorganisms, herbivores, and neighboring plants—that modulate biosynthetic pathways and the phenotypic expression of terpenoids (Ramos et al. 2023, 2020).

From an applied perspective, the identification of well-delimited chemotypes provides a framework for the rational selection of cultivars and cultivation sites for specific purposes. Monoterpene*-* and aroma-rich chemotypes may be preferable for applications in food flavorings and fragrances, whereas chemotypes rich in *E-*β-caryophyllene, α-humulene, and their oxidized derivatives are particularly promising for pharmaceutical, nutraceutical, and agrochemical uses, given the well-recognized bioactivity of these sesquiterpenes. Furthermore, extreme oxidative profiles and minor chemotypes, which are currently often relegated to by-products of the *Cannabis* industry, represent a still underexplored source of high-value*-*added terpenoids that can be harnessed within plant biorefinery chains.

**Fig. 4:**
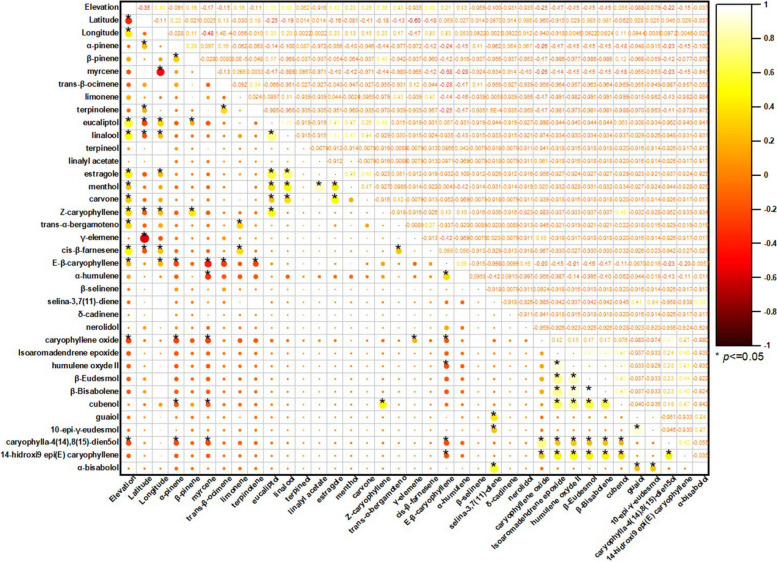
Correlation plot of terpenoid composition in VOs: Relationships with elevation across globally distributed samples. Legend: Red and yellow colors represent positive and negative correlations, respectively, with correlation coefficient indicated by both the color intensity and circle size, yellow circle (positive correlation), red circle (negative correlation)

The Pearson correlation matrix summarized in the correlogram (Fig. 4) describes the linear relationships between the 35 major terpenoids of the VOs and the geographical variables (elevation, latitude, and longitude). In the plot, circle color and diameter encode the sign and magnitude of the coefficients, respectively, whereas asterisks denote statistically significant correlations (*p* < 0.05).

When evaluated as an isolated predictor, elevation displays weak to moderate associations with the volatile composition, indicating limited explanatory power when considered alone. Menthol appears as an atypical constituent in the compiled dataset and is supported by a very limited number of reports; therefore, any patterns involving menthol should be regarded as exploratory and interpreted with caution**.** Within the oxygenated monoterpenes, elevation correlates positively, although with modest magnitude, with eucalyptol (*r* = 0.59; moderate; *p* < 0.0001), linalool (*r* = 0.48; moderate; *p* < 0.0001), and estragole (*r* = 0.43; moderate; *p* < 0.0001). Additional positive associations are also observed for carvone (*r* = 0.29; weak; *p* = 0.0008), *Z*-caryophyllene (*r* = 0.36; weak; *p* < 0.0001), *trans*-α-bergamotene (*r* = 0.33; weak; *p* = 0.0001), *cis*-β-farnesene (*r* = 0.48; moderate; *p* < 0.0001), and *E*-β-caryophyllene (*r* = 0.21; weak; *p* = 0.019). Conversely, elevation is negatively correlated with selected oxygenated sesquiterpenes, most notably caryophyllene oxide (*r* = –0.25; weak; *p* = 0.004) and caryophylla-4(14),8(15)-dien-5-ol (*r* = –0.22; weak; *p* = 0.012), whereas nerolidol shows a negligible and non-significant association (*r* = –0.03; *p* = 0.698). Collectively, these data indicate that elevation acts primarily as a fine modulator of volatile profiles, favoring slightly higher relative levels of oxygenated monoterpenes, but without inducing major restructuring in the overall chemical architecture of the oils.

In contrast, inter-compound correlations reveal a markedly more organized internal structure, consistent with coordinated metabolic regulation. Among oxygenated monoterpenes, a robust positive cluster is evident: eucalyptol–linalool shows a strong correlation (*r* = 0.76; *p* < 0.0001), while linalool–estragole remains strongly associated (*r* = 0.72; *p* < 0.0001) and eucalyptol–estragole exhibits a moderate-to-strong relationship (*r* = 0.61; *p* < 0.0001). A distinct co-accumulation pattern is observed for linalyl acetate (*r* = 0.87; *p* < 0.0001), which shows consistent co-variation with other recurrent oxygenated monoterpenes across the dataset. Carvone co-varies moderately with estragole (*r* = 0.48; *p* < 0.0001) and shows weaker-to-moderate linkages with eucalyptol (*r* = 0.26; *p* = 0.003) and linalool (*r* = 0.34; *p* < 0.0001), reinforcing the notion of structured co-occurrence among selected oxygenated monoterpenes.

The oxygenated sesquiterpenes also exhibit cohesive organization, but as submodules rather than a single homogeneous block. A prominent module is defined by isoaromadendrene epoxide, which correlates very strongly with humulene epoxide II (*r* = 0.90; *p* < 0.0001) and strongly with β-eudesmol (*r* = 0.77; *p* < 0.0001), β-bisabolene (*r* = 0.79; *p* < 0.0001), and cubenol (*r* = 0.66; *p* < 0.0001). Within this same module, β-eudesmol–β-bisabolene approaches a near-perfect association (*r* = 1.00; *p* < 0.0001), and both compounds remain strongly coupled to humulene epoxide II (0.86 ≤ *r* ≤ 0.88; *p* < 0.0001). A second, chemically coherent pair is observed for caryophyllene oxide–caryophylla-4(14),8(15)-dien-5-ol (*r* = 0.77; *p* < 0.0001), while guaiol forms a separate strong association with 10-*epi*-γ-eudesmol (*r* = 0.91; *p* < 0.0001), suggesting an additional branch of coordinated oxygenated sesquiterpene accumulation.

The correlations between hydrocarbon monoterpenes and several sesquiterpene-derived markers tend to be negative and of low-to-moderate magnitude, supporting an antagonistic chemical organization between monoterpene-centered profiles and sesquiterpene-enriched profiles. Representative examples include myrcene–*E*-β-caryophyllene (*r* = –0.38; *p* < 0.0001), myrcene–caryophyllene oxide (*r* = –0.26; *p* = 0.003), α-pinene–caryophyllene oxide (*r* = –0.26; *p* = 0.004), as well as negative associations with caryophylla-4(14),8(15)-dien-5-ol (*r* = –0.23; *p* = 0.01). Taken together, these patterns demonstrate that enrichment in particular chemical classes typically occurs in a concerted manner: monoterpene-driven aromatic signatures co-accumulate oxygenated monoterpenes (eucalyptol, linalool, estragole, carvone, and linalyl acetate), whereas sesquiterpene-enriched oils exhibit structured co-accumulation of oxygenated derivatives (isoaromadendrene epoxide, humulene epoxide II, β-eudesmol, β-bisabolene, cubenol) and additional specialized pairs (guaiol–10-*epi*-γ-eudesmol; caryophyllene oxide–caryophylla-4(14),8(15)-dien-5-ol). This architecture is consistent with shared biosynthetic and oxidative processes operating on hydrocarbon precursors, and supports the interpretation that *C. sativa* volatile profiles emerge from the combined influence of genetic determinants and environmental drivers that differentially reroute metabolic fluxes, resulting in pronounced compound-level variation.

Taken together, these correlation patterns demonstrate that enrichment in particular chemical classes tends to occur in a concerted manner: oils rich in oxygenated sesquiterpenes show co-accumulation of multiple caryophyllene/humulene derivatives (for example, isoaromadendrene epoxide, humulene epoxide II, β-eudesmol, cubenol, and guaiol) (Nigam et al. 1965; Ross and ElSohly 1996; Bertoli et al. 2010; Górski et al. 2016; Judžentienė et al. 2023; Smeriglio et al. 2020; Soares et al. 2023; Luca et al. 2024; Juliano et al. 2024), whereas oils with a monoterpene*-*based aromatic signature concentrate simultaneously several oxygenated monoterpenes (eucalyptol, linalool, estragole, linalyl acetate, and carvone) (Gorelick and Bernstein 2017; Silva Sofrás and Desimone 2022; Tricco et al. 2018; Chaiwangrach et al., 2025b; Cerrato et al. 2021). This organization is consistent with biosynthetic relationships —shared oxidative pathways from hydrocarbon precursors—and with oxidation processes occurring during drying and storage, particularly in the case of epoxides and sesquiterpene alcohols. Such evidence supports the view that the composition of *C. sativa* VOs is strongly modulated by a combination of genetic factors and biotic and abiotic environmental drivers, which reroute metabolic fluxes and result in marked variation in compound production.

### Regional scale—Italy focus

The published literature on *C. sativa* VO composition highlights marked regional chemical disparities. However, there remains a notable scarcity of studies in the Americas and Africa, and no records of such research in Oceania. This limited geographical representation can be attributed to a combination of factors. Legal restrictions on *C. sativa* cultivation and use in many countries continue to pose substantial barriers to research, and regulatory frameworks often differ widely. In several nations, permission to conduct *C. sativa* research has only recently been granted and is typically subject to stringent conditions. In regions where *C. sativa* has not historically been incorporated into agricultural systems, such as large parts of Africa and Oceania, there is also a lack of infrastructure, technical capacity, and dedicated funding for studies on this crop. Furthermore, local research agendas often prioritize long-established food or cash crops, which further limits the scope of investigations on *C. sativa*, particularly in countries where its use remains socially or politically controversial.

In contrast, Asia, and Europe with Italy as a prominent example, stand out as the most represented regions in studies of *C. sativa* VOs (Fig. 3; reference codes listed in Supplementary Table SS2). The higher number of scientific publications from these regions can be ascribed to more consolidated research frameworks, greater industrial interest in hemp- and *Cannabis*-derived products, and, in some cases, more permissive legal environments that actively encourage the systematic investigation of the plant’s chemical properties. These factors together create favorable conditions for sustained research, particularly in Europe, where countries such as Italy have emerged as key contributors to the field.

**Fig. 5:**
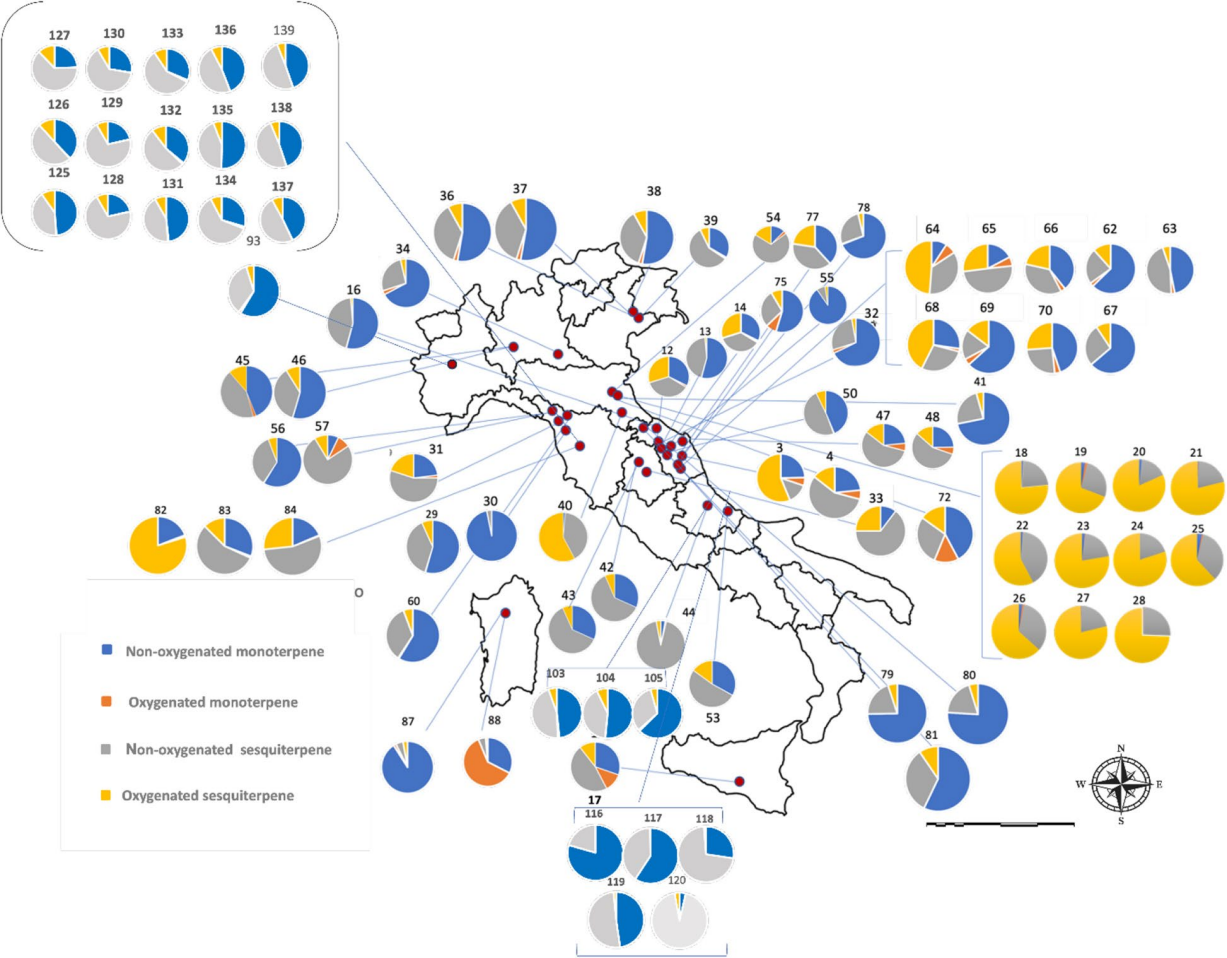
Geographical distribution of *C. sativa* chemotypes in Italy based on volatile*-*oil studies. Each pie chart represents one sampling site (*n* = 25 studies) and shows the relative contribution of non-oxygenated monoterpenes, oxygenated monoterpenes, non-oxygenated sesquiterpenes and oxygenated sesquiterpenes. Geographic coordinates and reference codes for each site are provided in Supplementary Table SS2

**Fig. 6:**
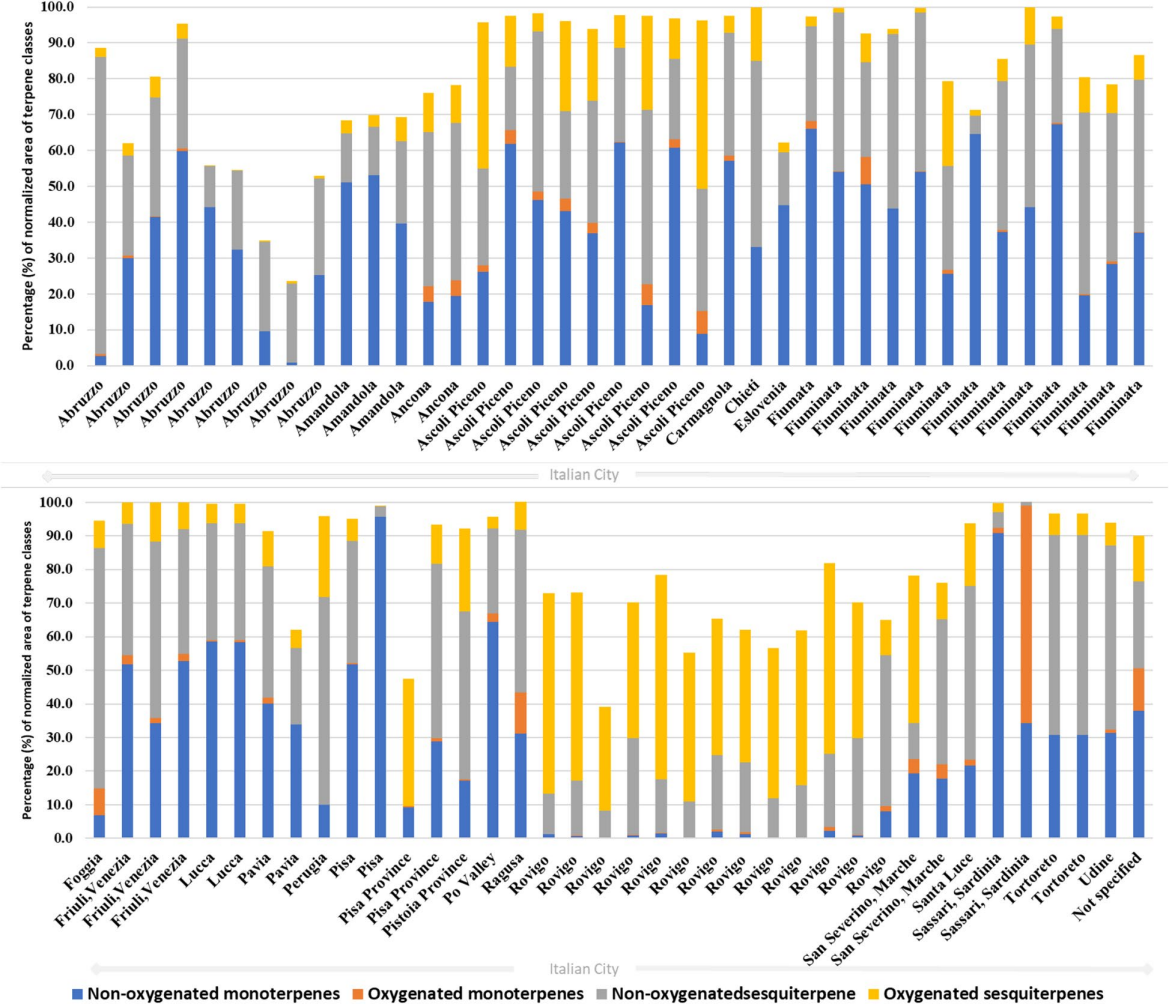
Distribution of terpenoid classes in volatile oils from *Cannabis* varieties cultivated in Italy. Stacked bar plots depict the percentage contribution of non-oxygenated monoterpenes, oxygenated monoterpenes, non-oxygenated sesquiterpenes and oxygenated sesquiterpenes for each city (25 Italian localities plus one sample with unspecified origin)

The VOs from cultivated *Cannabis* in Italy were classified according to the concentration of their major constituents (content > 5%; Supplementary Table SS1). The spatial distribution of chemotypes across Italy (Fig. 5), combined with the global variability in terpenoid classes (Fig. 2) and the relative contribution of monoterpenes and sesquiterpenes in each Italian locality (Fig. 6), shows that monoterpenoids and sesquiterpenoids constitute the dominant components of *C. sativa* VOs. A pronounced qualitative and quantitative contribution of non-oxygenated sesquiterpenes, particularly β-caryophyllene and α-humulene, was observed, in agreement with previous reports that identify these compounds as chemotaxonomic markers of the species (Jin et al. 2020; Cerrato et al. 2021). Localities such as Rovigo, San Severino Marche, and Ascoli Piceno exhibit unusually high levels of oxygenated sesquiterpenes for *C. sativa* cultivars, with total contents ranging from approximately 5–60% (Fig. 6). By contrast, VOs obtained from Pakistani *Cannabis* accessions show profiles enriched in oxygenated monoterpenes (Fig. 6), underscoring the marked phytochemical differentiation between European and Asian germplasm.

Across the Italian dataset, the most abundant individual compound was the bicyclic sesquiterpene β-caryophyllene, which predominated in 33 of the 39 samples analyzed, with concentrations ranging from 4.9% to 50.0%. β-Caryophyllene is widely used as a flavoring agent and has recognized anti-inflammatory, analgesic and antipyretic properties (Cerrato et al. 2021). Other major constituents include the aliphatic monoterpene β-myrcene, the bicyclic monoterpene α-pinene, the sesquiterpene α-humulene, caryophyllene oxide, and terpinolene. α-Humulene (8.7–9.1%) is also notable for its anti-inflammatory activity (Olejar and Park 2022). β-Myrcene (0.2–49.3%), biosynthesized by several plant species, particularly hops (*Humulus lupulus* L.), is widely employed in synthetic fragrances, pharmaceutical preparations, personal care products, and as a flavoring agent in foods (Adamek et al. 2022). α-Pinene (0.2–29.2%), detected in nearly all VOs, imparts a characteristic pine*-*resin odor and is used as a flavoring, solvent, and additive in lubricating oils (Adamek et al. 2022).

Profile analysis of VOs has been proposed as a complementary tool to distinguish dioecious from monoecious *C. sativa* genotypes, and as a chemical marker system to identify fiber*-*type varieties (Cerrato et al. 2021). Nevertheless, there is still a substantial gap in the literature regarding chemotypes associated with high levels of Δ^9^-THC and cannabigerol (CBG), as well as those enriched in less prevalent cannabinoids. This gap likely reflects regulatory restrictions on cultivars with Δ^9^-THC contents above 0.2%, coupled with the scarcity of studies focused on minor cannabinoids such as tetrahydrocannabivarin (THCV), cannabidivarin (CBDV) and cannabichromene (CBC). Most available work concentrates on genotypes that are legally approved in Europe, including dioecious varieties (*e.g.*, Fibrante, Fibranova, and Carmagnola) and monoecious cultivars (*e.g.*, Carmaleonte and Codimono), as well as hybrids derived from crosses between these lineages, such as 24 K, and Gorilla Glue (Table 1; *C. sativa* VOs, time*-*trend analysis).

**Table 1:** Biogeographic provinces and the cultivar of *Cannabis*. used in different countries

Biogeographic province/Country	Genotype (cultivar)	Reference
Amstetten, Austria	Fedrina 74, SwissMix,Kompolti, Secuemi	Novak et al. 2003)
Zurich, Switzerland	Férimon 12, Fédora 19, Félina 34, Futura 77, Kompolti, Kompolti hibrid TC, Uniko-B, FxT, Fibramulta 151, Trene, Lovrin 110, Secuieni 1, Livonie (landrace), Novosadska, Swissmix, Amtbol 398, B 3985 TE, Skunk	Mediavilla and Steinemann 1997)
San Severino Marche, Italy	Carmagnola Selezionata	Fiorini et al. 2020)
Kashimira, Pakistan	*Cannabis sativa* and *C. indica*	Naz et al. 2017)
Burgenland, Áustria	*Cannabis ruderalis* (wild)	Novak et al. 2003)
Rif, Marroc	-	Benelli et al. 2018)
Fiuminata, Italy	Felina 32	Benelli et al. 2018)
Ragusa, Italy	Futura 75, Felina 32, Kompolti, Carmagnola and Finola	Micalizzi et al. 2021b)
Rovigo, Italy	Uso-31, Carmaleonte, Codimono, Futura 75, Felina 32, Bernabeo, Carmagnola, Fibranova, Fibrante, Eletta Campana	Bertoli et al. 2010)
Pisa, Italy	Carmagnola, Carmagnola Selezionata, Red Petiole, Pop 1, Pop 2, Pop 3, Pop 4, Pop 5, Codimono and Felina 34	Bertoli et al. 2010)
Santa Luce, Italy	Fedora 17	Ascrizzi et al. 2019)
Fiumata, Italy	Felina 32, Carmagnola Selezionata	Rossi et al. 2020)
Perugia, Italy	Kompolti, Tisza	Di Sotto et al. 2022)
Po Valley, Italy	Carmagnola, Fibranova, Futura	Nissen et al. 2010)
Tenniken, Switzerland	Kompolti	Mediavilla and Steinemann 1998)
Friuli Venezia, Italy	Felina	Da Porto et al. 2014)
Udine, Italy	Fedora, Ferimon, Futura	Vuerich et al. 2019)
Rovigo, Italy	Uso-31, Carmaleonte, Codimono, Futura 75, Felina 32, Bernabeo, Carmagnola, Carmagnola Selezionata, Fibranova, Fibrante, Eletta Campana	Pieracci et al. 2021)
Eslovenia	Carmagnola, Tiborszallasi, Finola	Eržen et al. 2021)
Tortoreto, Italy	Futura	Pellegrini et al. 2021)
Abruzzo, Italy	Carmagnola, Kompolti, Futura 75, Gran Sasso Kush and Carmagnola Lemon	Palmieri et al. 2021)
Pavia, Italy	*Cannabis sativa* L. cv. Monoica	Gunjević et al. 2021)
Ancona, Italy	Carmagnola Selezionata	Bakali et al. 2022)
El Aiún, Marroc	Beldiya, Mexicana, Critical Plus	Bakali et al. 2022)
Fiuminata, Italy	Futura 75	Benelli et al. 2018)
Slovenia	Tiborszallasi, Futura 75	Laznik et al. 2020)
Chieti, Italy	Futura 75	Laznik et al. 2020)
Rovigo, Italy	Chinese accession (G-39); fibrante variety with low Δ^9^-THC content	Mazzara et al. 2022)
Fiuminata, Italy	Futura 75	Mazzara et al. 2022)
Lucca, Italy	Chinook, Kompolti	Ovidi et al. 2022)
Netherland	Bedrocan®	Ternelli et al. 2020)
Poznań, Poland	Beniko, Bialobrzeskie, Silesia	
Lucca, Italy	Chinook, Kompolti	Ovidi et al. 2022)
Brazil	-	Soares et al. 2023)
Ascoli Piceno, Italy	24 K, Gorilla Glue, Lemon, Conti Kush, Lemon Conti Kush New, Fresh Mountain, Amnesia Cookies, Pablito, White Shark and Venom OG	Ternelli et al. 2020)
Dublin, Ireland	Felina 32	Majidiyan et al. 2022)
Italy	-	Tognolini et al. 2006)
Backi Petrovac, Serbia	Carmagnola Selezionata, Spic, Helena, Carmagnola, Squieni, Bacalmas, Simba, Silesia, Chameleon, Fibrol, Futura, and Lovrin	Zheljazkov and Maggi 2021)
Kovacica, Serbia	Carmagnola Selezionata, Spic, Helena, Carmagnola, Squieni, Bacalmas, Simba, Silesia, Chameleon, Fibrol, Futura, and Lovrin	Zheljazkov and Maggi 2021)
Fiuminata, Italy	Felina 32	Fiorini et al. 2019)

Recent investigations demonstrate that post-harvest handling and processing exert a major influence on the chemical composition of VOs from *C. sativa* and *C. sativa* subsp. *indica*. Although extraction protocols can affect the total yield of volatiles, the qualitative profile (*i.e.*, which compounds are present) appears to be more sensitive to pre*-*harvest factors such as genotype, pedoclimatic conditions and harvest timing (Moreira et al. 2021). The drying of inflorescences, for example, has been associated with VOs enriched in sesquiterpenes (Kant et al. 2015). Comparative analyses of VOs from different geographical regions reveal pronounced variations in total sesquiterpene content: samples from Amstetten (72.8%) and Burgenland (32.5%) in Austria, Zürich (69.7%), and Tenniken (60.7%) in Switzerland, Slovenia (44.7%), the Netherlands (56.5%), and several Italian regions – Pisa (95.7%), Fiuminata (66.1%), Po Valley (64.4%), Pavia (40.1%), and Lucca (58.5%) – exemplify this variability. While sesquiterpene levels vary considerably between regions, extraction methods themselves have a comparatively modest impact on qualitative composition, indicating that growing conditions and chemotype exert stronger control over VO profiles (Torkamaneh and Jones 2022).

Drying is a critical step that affects both the quality and quantity of volatile constituents. Beyond their medicinal value, volatiles recovered from *C. sativa* biomass and residues have potential applications in pharmaceutical, food, cosmetic, and agrochemical industries, reinforcing the need to re*-*evaluate industrial hemp as an environmentally sustainable crop (Adamek et al. 2022). During drying, water loss and associated changes in cell integrity can alter the localization and stability of volatiles, resulting in compositional shifts (Campbell et al. 2019). For instance, in *Mentha aquatica* L., drying at 40 °C for 48 h increased menthol and menthone contents, whereas drying at 60 °C for the same duration led to their depletion (Adhikary et al. 2021). In general, higher drying temperatures tend to enhance the relative abundance of sesquiterpenes, although the magnitude and direction of these effects are compound- and species-dependent (Campbell et al. 2019).

Different drying techniques, including natural drying in sun or shade, hot-air oven drying, vacuum drying, microwave drying, and freeze*-*drying, transfer energy at distinct rates and over varying exposure times, thereby triggering different, often irreversible, chemical, and biological reactions. These processes also induce structural, physical, and mechanical changes in plant tissues. Consequently, it is essential to evaluate how each drying method affects final VOs quality (Thorpe 2006). In addition to intrinsic factors such as plant variety, pedoclimatic conditions and harvest stage, extrinsic factors including light exposure, oxygen availability, ambient humidity, and storage temperature can profoundly influence compound stability (Chandra et al. 2020). The deliberate choice of monoecious or dioecious varieties, together with careful control of field humidity and harvesting schedules, has been shown to improve VOs yield and concentration, increasing trichome density and terpene production (Cerrato et al. 2021).

In the context of post-harvest processing, extraction by solid-phase microextraction (SPME) has been reported to increase the monoterpene fraction from 51.7% to 95.7% in certain *C. sativa* samples (Jin et al. 2020; Nemati et al. 2021; Danziger and Bernstein 2021a). Grinding and drying steps also significantly affect VOs composition (Ramos 2019). Zheljazkov et al. (Zheljazkov et al. 2020) showed that grinding the plant material prior to distillation more than doubled the monoterpene content, with most of these compounds being recovered in the early stages of extraction. Conversely, the best conditions for preserving sesquiterpenes such as β-caryophyllene, α-humulene, α-bisabolol, and *E*-α-bergamotene were obtained when the material was not ground and distillation continued for 80 min, yielding an approximately 80% increase in total sesquiterpenes. This protocol is therefore particularly suitable for maximizing the utilisation of *C. sativa* residues. Similarly, Sellami et al. (Sellami et al. 2011) demonstrated that carefully controlled drying regimes can favor the retention of specific terpenes. Optimizing these processing parameters is thus crucial to ensure high and reproducible VO quality.

To further explore spatial and chemotypic patterns, multivariate analyses were performed on the Italian dataset compiled from published VO profiles of *C. sativa* (Bertoli et al. 2010; Da Porto et al. 2014; Fiorini et al. 2019; Pieracci et al. 2021; Smeriglio et al. 2020; Benelli et al. 2018; Mazzara et al. 2022, 2023; Juliano et al. 2024; Nissen et al. 2010; Barbalace et al. 2023; Ascrizzi et al. 2024; Spinozzi et al. 2025); (see Supplementary Table SS1 for the full list).

**Fig. 7:**
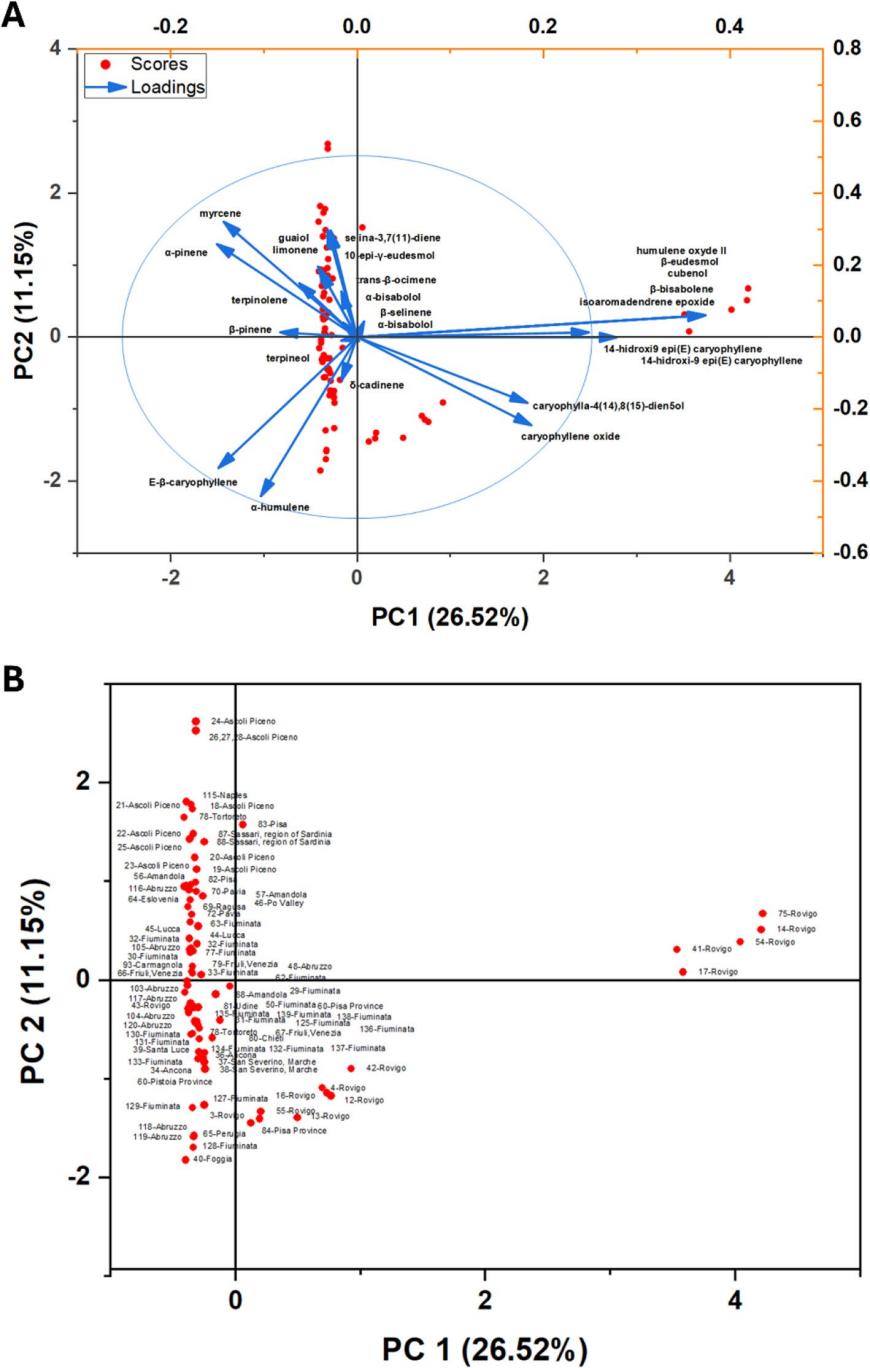
Principal component analysis (PCA) of volatile-oil composition in Italian *Cannabis* samples. (A) PCA biplot showing sample scores (red dots) and loading vectors (blue arrows) for the major monoterpene and sesquiterpene constituents. The 95% confidence ellipse for the score distribution is shown. (B) Score plot highlighting the dispersion of samples along PC1 and PC2. PC1 and PC2 account for 26.5% and 11.1% of the total variance, respectively. Reference codes for each site are provided in Supplementary Table SS2

PCA (Fig. 7) summarized the chemical variability of VOs obtained from Italian *Cannabis* samples collected across distinct regions. The first two principal components explained 26.52% (PC1) and 11.15% (PC2) of the total variance, respectively, accounting for 37.67% of the overall information captured by the model. In the biplot representation (Fig. 7A, and Supplementary Table SS4), the 95% confidence ellipse delimited the global dispersion of sample scores, showing that most samples remained concentrated around the central multivariate domain, whereas a restricted subset exhibited directional displacement associated with chemically distinctive signatures. Although the first two components explain a moderate fraction of the total variance, the ordination reveals substantial yet structured compositional heterogeneity across Italian localities, supporting the existence of recurrent chemotypic profiles rather than purely stochastic fluctuations in terpene composition.

PC1 represented the major axis of chemical differentiation and primarily described a gradient separating monoterpene-enriched profiles from compositions dominated by sesquiterpene derivatives and oxidation-associated markers within the caryophyllene/humulene block. On the positive side of PC1, the highest loading vectors included oxygenated/oxidized sesquiterpenes such as isoaromadendrene epoxide, humulene oxide II, β-eudesmol, cubenol, and bisabolol-related signatures, together with hydroxylated caryophyllene-type derivatives (notably 14-hydroxy-9-*epi*-(*E*)-caryophyllene-type compounds).

Caryophyllene oxide also displayed a relevant positive contribution along PC1 and an opposing (negative) component along PC2, consistent with its role as a marker of oxidative drift and chemotypic specialization along the oxidation axis (Bertoli et al. 2010; Pieracci et al. 2021; Smeriglio et al. 2020; Ascrizzi et al. 2024). In contrast, the negative side of PC1 was supported mainly by hydrocarbon monoterpenes, especially α-pinene, β-pinene, myrcene, limonene, trans-β-ocimene, and terpinolene, reflecting “fresh” monoterpene-rich chemistries commonly reported in several Italian case studies (Bertoli et al. 2010; Fiorini et al. 2019; Benelli et al. 2018; Mazzara et al. 2022; Barbalace et al. 2023; Ascrizzi et al. 2024). Importantly, within this negative PC1 domain, sesquiterpene hydrocarbons also contributed substantially, with *E*-β-caryophyllene and α-humulene showing strong weights and marking a second, structurally coherent block associated with sesquiterpene hydrocarbon dominance. Accordingly, PC1 can be interpreted as the principal compositional trajectory spanning (1) monoterpene-rich oils and (2) sesquiterpene-dominated oils shifting towards oxygenated/oxidized derivatives, reflecting both biosynthetic emphasis and potential post-harvest oxidative conversion dynamics.

PC2 described a secondary gradient refining the discrimination among samples already positioned along PC1. Positive PC2 values were preferentially associated with compounds such as myrcene, α-pinene, selina-3,7(11)-diene, guaiol, and 10-*epi*-γ-eudesmol, indicating that this axis integrates both monoterpene abundance (particularly myrcene/α-pinene) and selective contributions of oxygenated sesquiterpenes (*e.g.*, guaiol- and eudesmol-related alcohols). Conversely, negative PC2 scores were strongly aligned with α-humulene and *E*-β-caryophyllene, and additionally with oxidation-associated markers exhibiting negative PC2 contributions (*e.g.*, caryophyllene oxide and caryophylla-4(14),8(15)-dien-5-ol). This arrangement indicates that PC2 operates as a refinement axis discriminating among sesquiterpene hydrocarbon-rich samples and those combining monoterpene dominance with targeted sesquiterpene oxygenation. In practical terms, PC2 enhances within-group resolution by capturing the balance between (1) caryophyllene/humulene hydrocarbon abundance and (2) mixed monoterpene–oxygenated sesquiterpene signatures, a pattern compatible with modulation by genotype, pedoclimatic conditions, agronomic management, and/or post-harvest processing (Fiorini et al. 2019; Benelli et al. 2018; Mazzara et al. 2022; Barbalace et al. 2023).

Inspection of sample scores (Fig. 7B) revealed partial geographic segregation, with Rovigo representing the clearest outlying behavior. Several Rovigo samples were markedly displaced towards strongly positive PC1 values, including extreme scores (*e.g*., 75-Rovigo, 14-Rovigo, 54-Rovigo, 17-Rovigo, 41-Rovigo), consistent with their association with oxygenated/oxidized markers and hydroxylated caryophyllene-related derivatives contributing positively to PC1 (Pieracci et al. 2021; Smeriglio et al. 2020; Ascrizzi et al. 2024). This separation supports the interpretation that a subset of Rovigo oils is chemically specialized and/or affected by enhanced oxidative conversion of sesquiterpene hydrocarbons. By contrast, most other localities remained concentrated at negative PC1 values, exhibiting extensive overlap among regions and a more compact distribution, in line with profiles dominated by hydrocarbon monoterpenes and/or sesquiterpene hydrocarbons such as *E*-β-caryophyllene and α-humulene. Along PC2, samples exhibiting higher positive values (*e.g.,* 24-Ascoli Piceno and 115-Naples) aligned more strongly with the myrcene/α-pinene/guaiol/eudesmol-associated vectors, whereas markedly negative PC2 scores (*e.g.,* 40-Foggia and several Fiuminata individuals) aligned with the α-humulene/*E*-β-caryophyllene direction and with oxidation-associated markers displaying negative PC2 contributions.

Collectively, the PCA indicates that volatile-oil variability in Italian *Cannabis* is primarily organized along an oxidation-associated transition axis (PC1) opposing monoterpene-rich signatures to oxygenated/oxidized sesquiterpene derivatives, while a second axis (PC2) structures additional differences in the relative prominence of sesquiterpene hydrocarbons versus mixed profiles enriched in myrcene and selected oxygenated sesquiterpenes. This multivariate architecture is consistent with regional chemovariation and/or differential modulation of terpene metabolism by environmental and agronomic drivers, with the strongest separation being observed for part of the Rovigo set along the positive PC1 domain.

In chemical biogeography, the existence of an indicator of a “chemical geotype” (chemical phenotypic plasticity arising from environmental conditions at a local scale) is often invoked to support interpretation (Ramos et al. 2023). However, the Italian PCA shows that regional labels do not yield a single, fully segregated chemotype; instead, they reflect a scenario of high chemical phenoplasticity, highlighting the potential of local environmental conditions to shape a geotype. Rovigo samples illustrate this pattern clearly: while a subset shifts strongly toward positive PC1 (e.g., 14-, 41-, 54-, and 74-Rovigo), other Rovigo entries remain embedded within the main multiregional cluster.

The loadings indicate that PC1 is driven primarily by oxygenated sesquiterpenes and by caryophyllene/humulene derivatives, suggesting that the “Rovigo-shifted” subset expresses a more oxidized/oxygenated signature. This within-locality dispersion supports a model in which chemotypes are shaped by both genetic background and environmentally modulated expression (phenotypic plasticity), as well as by anthropogenic influences, potentially compounded by methodological differences among studies (extraction and post-harvest handling), rather than being determined solely by geographic origin.

**Fig. 8:**
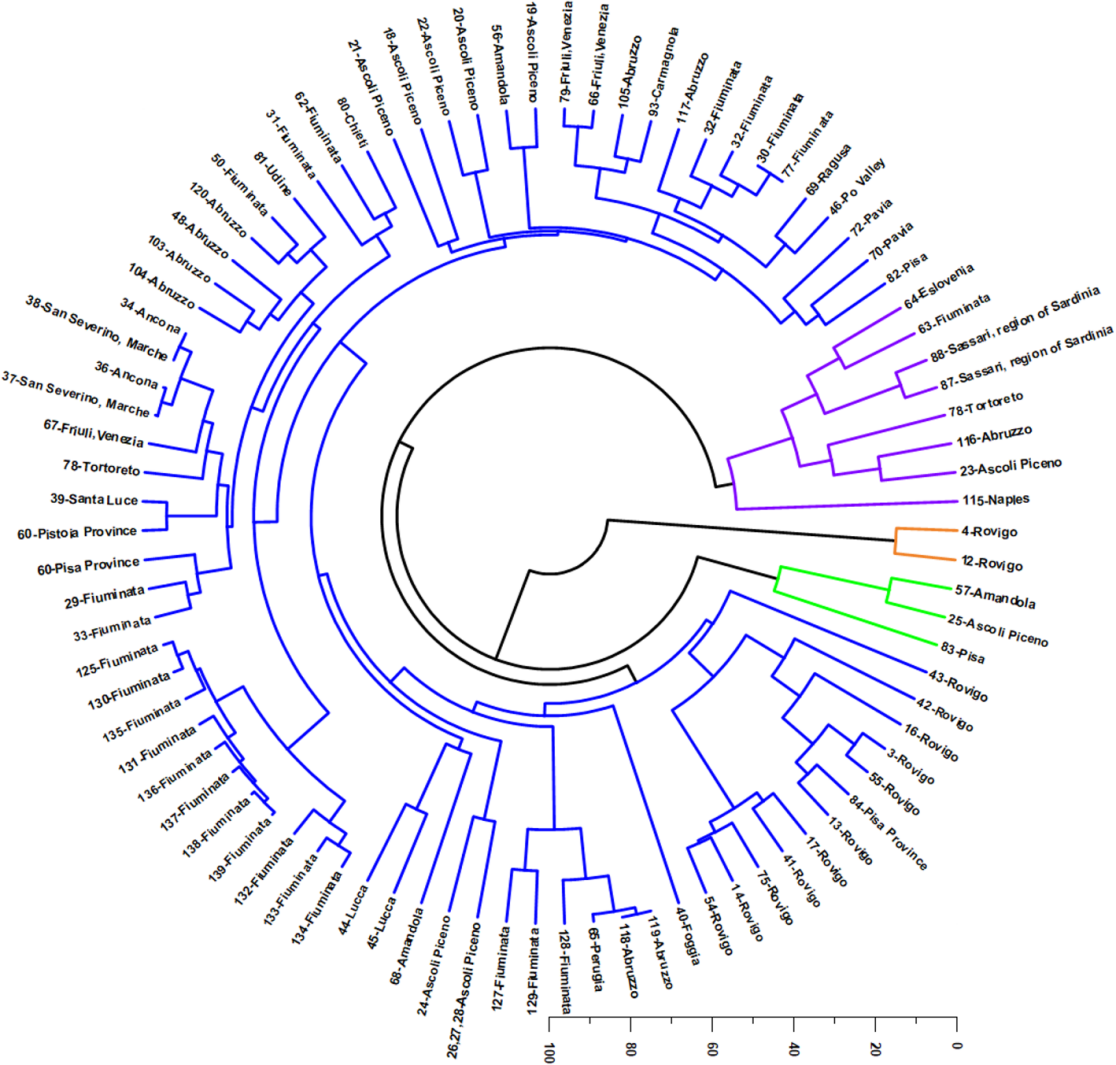
Hierarchical Cluster Analysis (HCA) of Italian *Cannabis* volatile-oil samples based on the relative abundance of 25 major terpenoids (≥ 5% in at least one sample).. The circular dendrogram resolves four main chemotypic clusters (color-coded): (i) a sesquiterpene hydrocarbon profile dominated by *E*-β-caryophyllene and α-humulene (blue); (ii) a monoterpene-rich profile characterized by myrcene and α-pinene, with relevant contributions of limonene and β-pinene (purple); (iii) an oxidized sesquiterpene profile dominated by caryophyllene oxide and caryophylla-4(14),8(15)-dien-5-ol (orange); and (iv) a terpinolene-dominant monoterpene profile (green). Reference codes for each site are provided in Supplementary Table SS2

HCA (Fig. 8) corroborated the PCA patterning and resolved four major chemotypic clusters, reinforcing the existence of discrete recurrent volatile-oil profiles. In contrast to earlier classifications proposing a higher number of clusters, the circular dendrogram supports a more parsimonious chemotypic structure, defined by four dominant compositional regimes (color-coded in Fig. 8): (1) a sesquiterpene hydrocarbon profile dominated by *E*-β-caryophyllene and α-humulene (blue); (2) a monoterpene-rich profile characterized by myrcene and α-pinene, with relevant contributions of limonene and β-pinene (purple); (3) an oxidized/oxygenated sesquiterpene profile dominated by caryophyllene oxide and caryophylla-4(14),8(15)-dien-5-ol (orange); and (4) a terpinolene-dominant monoterpene profile (green). Thus, considering the major constituents driving sample separation, three structural blocks remain mechanistically decisive hydrocarbon monoterpenes, sesquiterpene hydrocarbons, and oxygenated/oxidized sesquiterpenes, yet the updated clustering indicates that monoterpene-rich profiles subdivide into two chemically meaningful subtypes: a myrcene/α-pinene profile versus a terpinolene-dominant profile.

The largest chemotypic block recovered by HCA (blue branches, Fig. 8) corresponds to a sesquiterpene hydrocarbon-dominated chemotype, defined by high mean contributions of *E*-β-caryophyllene and α-humulene. This cluster reflects oils with a clear sesquiterpene hydrocarbon backbone, typically accompanied by secondary monoterpene contributions (*e.g.*, α-pinene and myrcene) but without monoterpenes becoming dominant. From a biosynthetic standpoint, this profile is consistent with strong flux through the farnesyl diphosphate pathway and preferential accumulation of caryophyllene/humulene congeners, a pattern repeatedly reported as a stable hallmark in multiple Italian localities and cultivation contexts (Da Porto et al. 2014; Fiorini et al. 2019, 2020; Benelli et al. 2018; Mazzara et al. 2022, 2023; Aguzzi et al. 2023; Ovidi et al. 2022; Juliano et al. 2024; Vinciguerra et al. 2024; Nissen et al. 2010; Micalizzi et al. 2021b).

In practical terms, this chemotype occupies the negative-to-intermediate PC1 domain while projecting towards negative PC2 values, reflecting the alignment of α-humulene and *E*-β-caryophyllene with the negative direction of PC2. This cluster may represent a baseline “caryophyllene–humulene” signature that is relatively resilient to monoterpene fluctuations, potentially reflecting genetic stability and/or cultivation conditions favoring sesquiterpene hydrocarbon production.

A second major cluster (purple branches) corresponds to a monoterpene-rich chemotype dominated by myrcene and α-pinene, with consistent secondary enrichment in limonene (≈ 8.4%) and β-pinene. Chemically, this profile reflects a pronounced monoterpene imprint (“resinous/pine-like” with citrus accents), where hydrocarbon monoterpenes overwhelmingly structure the aromatic identity and the sesquiterpene fraction becomes quantitatively subordinate.

This pattern aligns with negative PC1 values and positive PC2 contributions, coherently reflecting the loading vectors of myrcene, α-pinene, and limonene. Within this monoterpene-rich cluster, an internal subgroup is evident for Sassari (Namdar et al. 2018; Georgieva and Kosev 2018), which tends to separate due to extremely high myrcene levels combined with comparatively lower α-pinene. By contrast, the remaining samples in the purple cluster exhibit a more balanced α-pinene/myrcene relationship (approximately a 1:1 ratio), supporting the interpretation of a geographically influenced substructure within the monoterpene domain (Juliano et al. 2024; Vinciguerra et al. 2024; Menghini et al. 2021; Pellegrini et al. 2021). This Sassari-associated deviation is compatible with insular Mediterranean conditions and/or cultivar-specific effects, reinforcing that monoterpene dominance can occur through alternative “myrcene-driven” or “balanced α-pinene/myrcene” approximately within the same broad chemotype.

The third cluster (orange branches) defines a highly specialized oxidized/oxygenated sesquiterpene chemotype, strongly isolated by the dominance of caryophyllene oxide and caryophylla-4(14),8(15)-dien-5-ol, with the volatile signature being essentially concentrated into these two constituents. In the dendrogram, this profile is most clearly represented by a restricted Rovigo subset (notably 4-Rovigo and 12-Rovigo), indicating that the most extreme oxidation-dominated phenotype corresponds to a narrowly defined chemotypic enclave rather than encompassing the entirety of the Rovigo material. This configuration is nevertheless congruent with the strong positive PC1 loadings of oxygenated/oxidized sesquiterpenes and hydroxylated caryophyllene derivatives (Pieracci et al. 2021; Smeriglio et al. 2020; Ascrizzi et al. 2024), and with the displacement of part of the Rovigo set towards positive PC1 values in the PCA.

From a mechanistic standpoint, this profile can be interpreted through two non-mutually exclusive hypotheses. First, it may represent genetically distinct material with enhanced intrinsic capacity for sesquiterpene oxidation and accumulation of oxygenated derivatives. Second, it may reflect pronounced post-harvest oxidative conversion of sesquiterpene hydrocarbons into oxygenated congeners, driven by storage conditions, drying parameters, time, oxygen exposure, and temperature. Notably, the overwhelming predominance of caryophyllene oxide strongly supports an oxidative route, consistent with the well-documented susceptibility of sesquiterpene hydrocarbons to conversion into epoxides and alcohol derivatives under oxidative environments (Bertoli et al. 2010; Pieracci et al. 2021; Smeriglio et al. 2020; Ascrizzi et al. 2024). Regardless of the driving mechanism, the orange cluster represents the most chemically distinctive phenotype recovered in the Italian dataset, functioning as a robust marker group with high discriminatory power.

The fourth cluster (green branches) corresponds to a terpinolene-dominant monoterpene chemotype, characterized by high terpinolene and substantial myrcene, accompanied by secondary *E*-β-caryophyllene and *trans*-β-ocimene. This cluster differs from the broader monoterpene-rich purple chemotype by the clear “terpinolene peak” structuring the profile, defining a chemically coherent subtype within monoterpene dominance. This group is geographically represented by a restricted subset of samples, including Pisa, Amandola, and Ascoli Piceno, in agreement with the partial segregation observed in PCA and supporting earlier regional associations for the Tuscany/Marche belt (Bertoli et al. 2010; Mazzara et al. 2022; Barbalace et al. 2023; Ascrizzi et al. 2024). Moreover, an internal subpattern is evident within the green cluster: the individual 83-Pisa exhibits unusually elevated trans-β-ocimene, whereas the remaining green members (25-Ascoli Piceno and 57-Amandola) show higher limonene and greater contribution of *E*-β-caryophyllene. This indicates that, even within the terpinolene-dominant regime, secondary monoterpenes and the sesquiterpene backbone modulate the final aromatic implementation, suggesting either cultivar effects or fine-scale environmental drivers acting on monoterpene branching pathways.

Taken together, the integrated PCA–HCA framework supports a chemically meaningful and internally consistent classification of Italian *Cannabis* VOs into four major chemotypes: (1) a sesquiterpene hydrocarbon *E*-β-caryophyllene/α-humulene type (blue), (2) a monoterpene-rich myrcene/α-pinene type with limonene/β-pinene contributions (purple), (3) an oxidized/oxygenated sesquiterpene type dominated by caryophyllene oxide and caryophylla-4(14),8(15)-dien-5-ol (orange), and (4) a terpinolene-dominant monoterpene type (green). Importantly, this updated structure refines previous interpretations by separating monoterpene-rich oils into two distinct regimes (myrcene/α-pinene versus terpinolene-driven), while preserving the biological and practical relevance of the sesquiterpene hydrocarbon and oxidized sesquiterpene extremes. Such multivariate convergence strongly indicates that regional Italian volatile-oil diversity is organized around a limited set of recurrent terpene design principles, plausibly shaped by genotype-by-environment interaction, cultivation conditions, and the chemical lability of sesquiterpene hydrocarbons toward oxidation during post-harvest handling (Bertoli et al. 2010; Fiorini et al. 2019; Benelli et al. 2018; Mazzara et al. 2022; Barbalace et al. 2023; Ascrizzi et al. 2024).

**Fig. 9:**
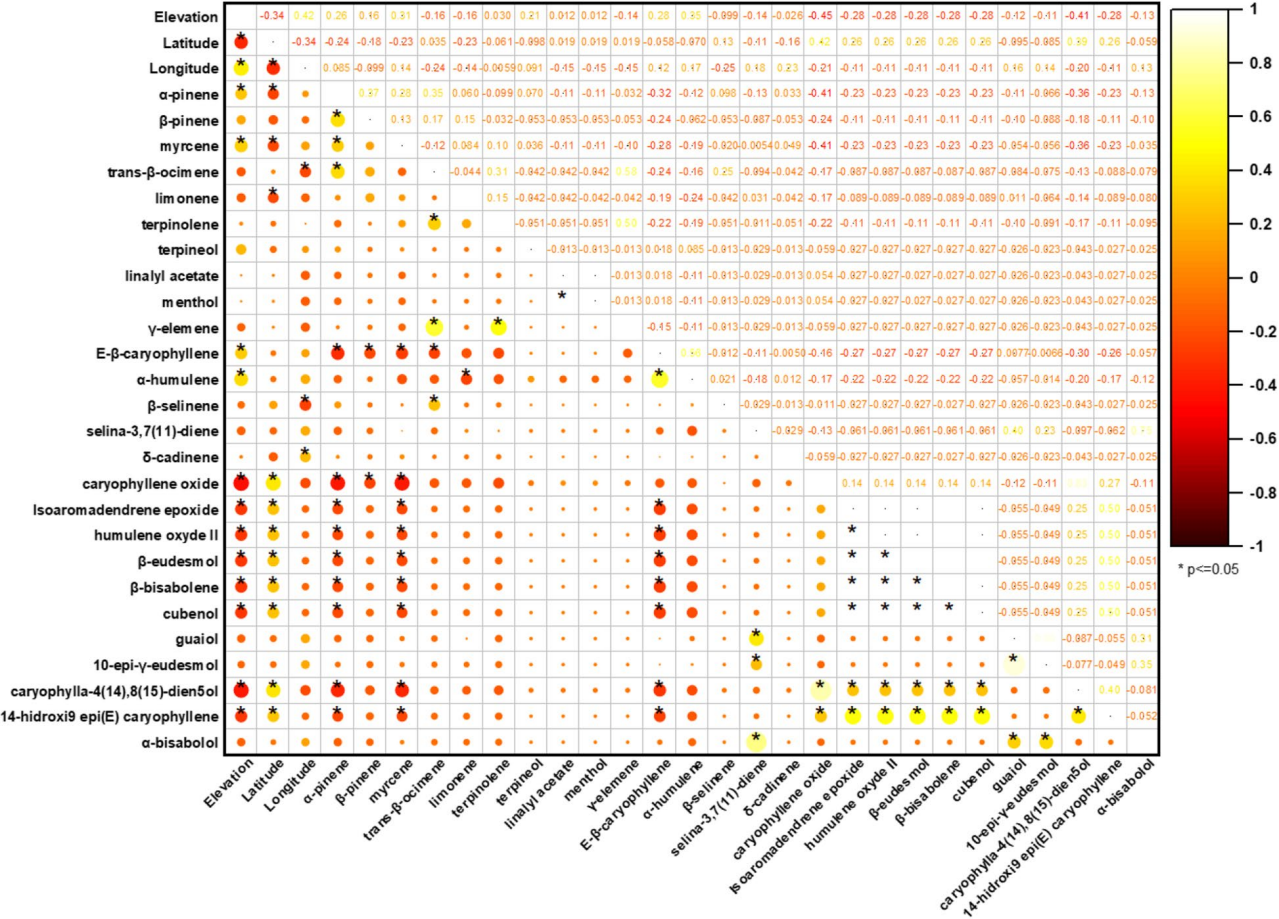
Correlation plot of terpenoid composition in VOs: relationships with elevation across samples distributed in Italy. Legend: Red and yellow colors represent positive and negative correlations, respectively, with correlation coefficient indicated by both the color intensity and circle size, yellow circle (positive correlation), red circle (negative correlation)

Pearson’s correlation analysis between geography and VO composition (Fig. 9) provides further support for these chemotypic patterns and was calculated on the same integrated dataset (Bertoli et al. 2010; Da Porto et al. 2014; Fiorini et al. 2019, 2020; Pieracci et al. 2021; Smeriglio et al. 2020; Benelli et al. 2018; Mazzara et al. 2022, 2023; Ascrizzi et al. 2024; Spinozzi et al. 2025). Elevation is negatively correlated with latitude (r = − 0.34, *p* < 0.05) and positively with longitude (r = 0.42, *p* < 0.05), reflecting a spatial gradient in which higher-altitude sites are located predominantly in central Apennine regions, whereas low-lying areas correspond to northern plains such as the Po Valley and Rovigo. Elevation shows positive correlations with classical monoterpenes (α-pinene, myrcene; r = 0.26–0.31, *p* < 0.05) and with non-oxygenated sesquiterpenes (*E-*β-caryophyllene, α-humulene; r up to 0.35, *p* < 0.05), but negative correlations with several oxygenated sesquiterpenes, including caryophyllene oxide and related epoxides and alcohols (r = − 0.28 to − 0.45, *p* < 0.05). Conversely, latitude is negatively associated with monoterpenes (α-pinene, myrcene, limonene; r = − 0.23 to − 0.24, *p* < 0.05) and positively correlated with oxygenated sesquiterpenes such as caryophyllene oxide, humulene oxide II, β-eudesmol, and cubenol (r = 0.26–0.42, *p* < 0.05). Thus, higher-altitude Apennine sites tend to accumulate monoterpenes and non-oxygenated sesquiterpenes, whereas low-altitude northern localities are enriched in oxidized sesquiterpenes.

The internal structure of the correlation matrix also reveals distinct chemical modules. Monoterpenes covary positively (for example α-pinene with β-pinene, and myrcene), defining a coherent monoterpenic block associated with the α-pinene/myrcene chemotype described for several central and southern localities (Fiorini et al. 2019; Benelli et al. 2018; Juliano et al. 2024; Palmieri et al. 2021). Non-oxygenated sesquiterpenes *E-*β-caryophyllene and α-humulene are strongly correlated (r = 0.56, *p* < 0.05), reflecting their shared biosynthetic origin and their joint occurrence in many Apennine samples (Mazzara et al. 2022, 2023; Fiorini et al. 2020). Oxygenated sesquiterpenes form an exceptionally cohesive “oxidative” module: caryophyllene oxide, isoaromadendrene epoxide, humulene oxide II, β-eudesmol, β-bisabolene, guaiol, 10-*epi*-γ-eudesmol, and α-bisabolol are all highly and positively intercorrelated (many pairs with r ≥ 0.8, *p* < 0.05), mirroring the strongly oxidized profiles documented for Rovigo and related samplesn (Pieracci et al. 2021; Smeriglio et al. 2020). Importantly, this oxidative module is antagonistic to the monoterpenic block, as indicated by significant negative correlations between α-pinene or myrcene and caryophyllene oxide or caryophylla-dienol (r = − 0.36 to − 0.41, *p* < 0.05). When monoterpenes dominate, oxygenated sesquiterpenes are consistently reduced, and vice versa.

At the regional scale, these gradients translate into clear geographic patterns. Low-altitude northern plains (Rovigo, Po Valley, and part of Friuli Venezia) are characterized by chemotypes rich in oxygenated sesquiterpenes, consistent with low elevation, high latitude, and the strong internal coherence of the oxidative module (Da Porto et al. 2014; Pieracci et al. 2021; Smeriglio et al. 2020; Nissen et al. 2010). In contrast, central Apennine and central–northern regions (Fiuminata, Ascoli Piceno, Amandola, San Severino, mountainous Abruzzo, parts of Tuscany and Umbria) show higher elevations and intermediate latitudes, favoring monoterpenes and non-oxygenated sesquiterpenes and aligning with the α-pinene/myrcene/caryophyllene–humulene chemotypes (blue and yellow clusters in Fig. 8) (Fiorini et al. 2019; Benelli et al. 2018; Zheljazkov et al. 2020; Aguzzi et al. 2023; Palmieri et al. 2021; Barbalace et al. 2023; Spinozzi et al. 2025; Micalizzi et al. 2021b; Di Sotto et al. 2022; Menghini et al. 2021). The central–eastern belt (Pisa, Amandola, part of Ascoli Piceno) hosts the citrus/terpinolene chemotype, occupying an intermediate position between monoterpenic and oxidized sesquiterpenic extremes (Bertoli et al. 2010; Mazzara et al. 2022; Barbalace et al. 2023). Southern and insular regions (Naples, Ragusa, Foggia, Sassari) generally present higher relative levels of monoterpenes and lower proportions of oxidized sesquiterpenes, in agreement with their lower latitudes and more Mediterranean climatic conditions (Ovidi et al. 2022; Juliano et al. 2024; Vinciguerra et al. 2024; Micalizzi et al. 2021b).

Taken together, the mapping of chemotypes (Figs. 5–6), multivariate analyses (Figs. 7–8) and correlation structure (Fig. 9), derived from the integrated analysis of Italian *C. sativa* VOs reported across more than twenty-five independent studies (Bertoli et al. 2010; Da Porto et al. 2014; Fiorini et al. 2019, 2020; Pieracci et al. 2021; Smeriglio et al. 2020; Benelli et al. 2018; Mazzara et al. 2022, 2023; Juliano et al. 2024; Nissen et al. 2010; Barbalace et al. 2023; Ascrizzi et al. 2024; Spinozzi et al. 2025), and references therein, depict Italian *C. sativa* inflorescences as a mosaic of volatile profiles organized along two main axes: (i) an environmental gradient (elevation/latitude) that opposes monoterpene*-* and non-oxygenated sesquiterpene *-*rich oils to oxygenated sesquiterpene*-*rich oils, and (ii) an internal chemical axis that structures compounds into tightly covarying modules (α-pinene/myrcene, limonene/terpinolene, caryophyllene/humulene, and oxidized caryophyllene/humulene derivatives). This mosaic reflects the combined influence of genotype, local environment, and post-harvest management and emphasizes the importance of territoriality in shaping the chemodiversity and potential bioactivity of *Cannabis* VOs.

### Stable or not stable is the question? Chemical phenotypic expression or plasticity control for quality requirement

Understanding the natural mechanisms underlying the qualitative and quantitative variation in the metabolic and chemical profiles of *Cannabis* specimens its chemical phenotypes is essential to explain why this species produces certain metabolites and how they interact. Insights from other species containing phytocannabinoids, such as *Helichrysum umbraculigerum* Less. (Pollastro et al. 2017), may also contribute to this understanding. Equally important is clarifying how different genotypes, environmental conditions, and biogeographic contexts influence biosynthetic pathways and phenotypic plasticity in *C Cannabis*-grown outdoors. Optimizing indoor cultivation by controlling environmental factors to obtain specific chemotypes, assessing chemical stability across different levels of chemodiversity (α, β, and γ), and developing targeted breeding programs may help explain the biochemical logic behind metabolite production and association.

Evidence*-*based cultivation practices are essential for producing more productive and chemically stable cultivars, not only of *Cannabis* but also of other medicinal plants of economic interest (Gorelick and Bernstein 2017). Achieving this goal requires integrating knowledge of phenotypic plasticity and chemical heritability to ensure genetic consistency and success in vegetative propagation (Campbell et al. 2019), Continuous propagation under cultivation, however, can lead to a gradual loss of chemical diversity (Ramos et al. 2022).

Although the morphological variability of *Cannabis* and its chemical composition have been less explored, both are likely related, given the documented qualitative and quantitative chemical variation during plant development and among different organs (Towler and Weathers 2015). Considering plant chemistry and its functional roles, it is also crucial to reflect on the technological processes that determine product quality (Moreira et al. 2021; Massuela et al. 2022; Laznik et al. 2020; Bar and Shtein 2019). For instance, the removal of fan or inflorescence leaves during extraction raises questions: what compounds are discarded, and how do they affect the final product? Mechanical clipping, often used to simulate herbivory, can itself induce qualitative and quantitative chemical changes (Kant et al. 2015). Furthermore, the quality, stability (Jin et al. 2020) and bioactivity of final products depend not only on cannabinoids but also on other bioactive compounds and their complex receptor interactions across the plant’s diverse chemovars (Cerrato et al. 2021).

Among the factors affecting chemical stability, one of the most important is cross-pollination in commercial crops combined with clonal propagation (Olejar and Park 2022; Adamek et al. 2022). This process strongly influences phenotype characterization, particularly in distinguishing chemotypes and genotypes with respect to sexual and clonal heritability (Ramos et al. 2022). Maintaining known genetic lines helps prevent declines in chemical vigor, biomass, and chemodiversity, and avoids unpredictable chemical fluctuations over time (Adamek et al. 2022; Ramos et al. 2022). Somatic mutations and epigenetic changes may also contribute to the pronounced chemical variability observed in clonal propagation. Yet, few studies have examined these phenomena in relation to spatiotemporal chemical variation across tissues, organs, and cultivars. This gap highlights the need for further research extending beyond cannabinoids to include other metabolites (Adamek et al. 2022; Torkamaneh and Jones 2022).

During cross-pollination, multiple factors can influence cannabinoid variation (Olejar and Park 2022). Understanding these processes will aid the *Cannabis* industry in defining best practices for producing high-quality genotypes, whether seeds, propagules, or mother plants, thereby minimizing mutation accumulation or propagation delays (Adamek et al. 2022; Torkamaneh and Jones 2022; Ramos et al. 2022). However, several challenges remain in obtaining elite cultivars: (1) identifying the optimal propagation matrix (Adamek et al. 2022; Campbell et al. 2019; Adhikary et al. 2021); (2) selecting seeds and propagation types suited to the cultivation purpose (Walker 2021); (3) choosing appropriate cultivation systems (indoor vs. outdoor) (Chandra et al. 2020); (4) management strategies and cultural treatments (Nemati et al. 2021); and (5) harvest standardization (Danziger and Bernstein 2021b). Indicators of quality assurance can be assessed through variations in: (1) local climatic conditions (Ramos et al. 2020; Ramos 2019); (2) macro- and microscopic morphometry; (3) genetics (Campbell et al. 2019; Burgel 2021); (4) biomass (Danziger and Bernstein 2021b; Namdar et al. 2018), and (5) resin yields. Other factors directly related to chemical stability must also be considered when choosing methods, such as (6) energy expenditure associated with different cultivation types, and (7) greenhouse gas emission (Torkamaneh and Jones 2022; Georgieva and Kosev 2018).

In the contexts of stability and phenotypic expression, we suggest that the lack of knowledge necessary for applied aspects is probably due to insufficient data on the ecological and evolutionary aspects that have shaped the current patterns of occurrence and abundance of secondary metabolites in *Cannabis*. Specifically, there is a lack of chemical ecology studies on *Cannabis* species and specimens.

### Integration and complementarity of cannabinoid and terpenoid biosynthetic pathways

**Fig. 10:**
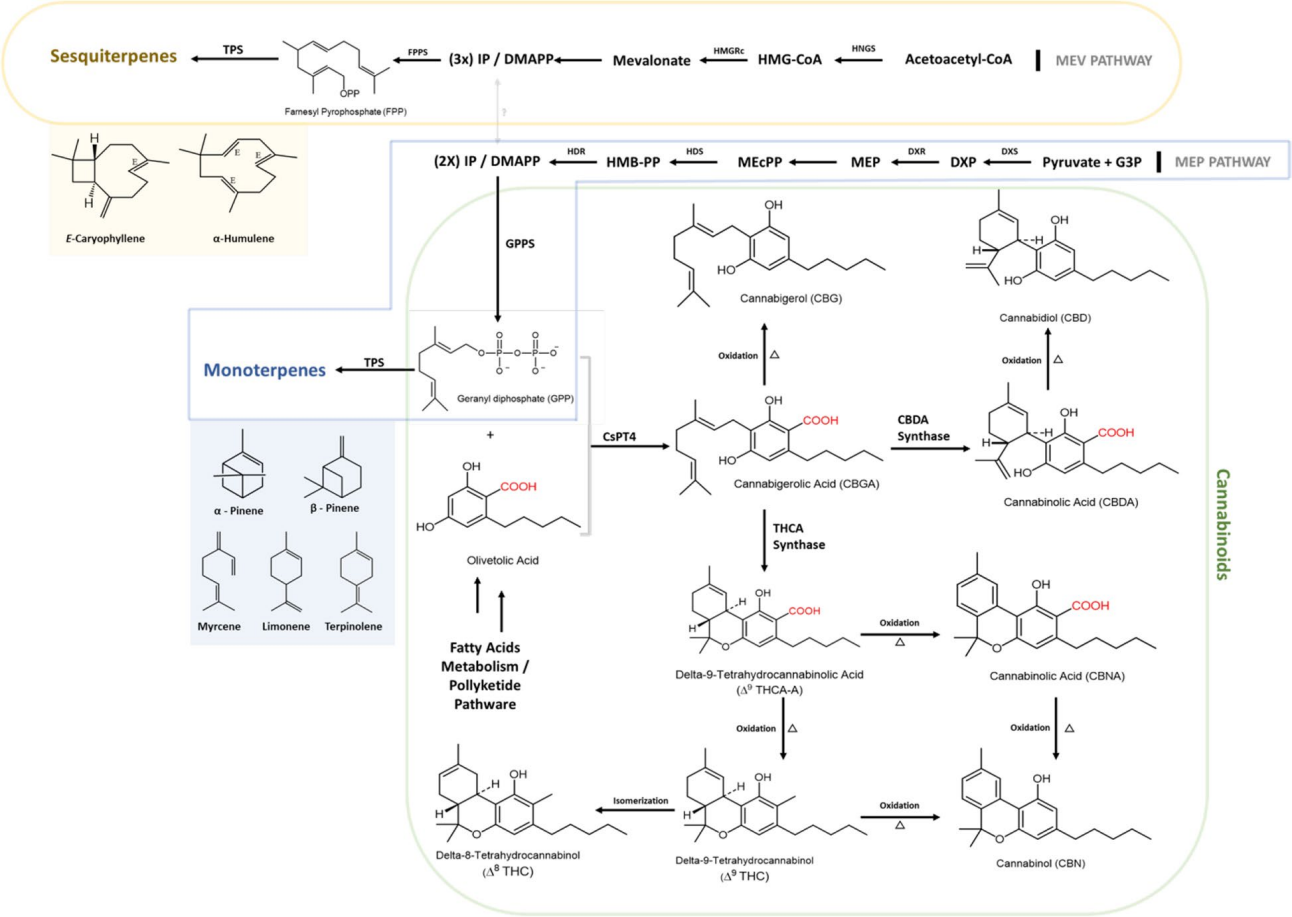
Integrated biosynthetic network of terpenoids and major acidic cannabinoids in *Cannabis sativa*. Plastidial MEP pathway–derived geranyl diphosphate (GPP) is partitioned between monoterpene biosynthesis (α-pinene, β-pinene, myrcene, limonene, terpinolene) and CsPT4-mediated prenylation of olivetolic acid to form cannabigerolic acid (CBGA), the central precursor of the principal acidic cannabinoids (*e.g.*, THCA, s, CBCA), whereas cytosolic MEV pathway intermediates give rise to sesquiterpenes such as β-caryophyllene and α-humulene; a putative metabolic crosstalk between the MEV and MEP pathways is indicated. Legends: MEV, mevalonate pathway; MEP, methylerythritol phosphate pathway; CsPT4, *C. sativa* prenyltransferase 4; GPP, geranyl diphosphate; CBGA, cannabigerolic acid; IP, isopentenyl pyrophosphate; DMAP, dimethylallyl pyrophosphate.? – The dynamics of IP/DMAP availability at the interface of the cannabinoid and terpenoid biosynthetic pathways remains partially elucidated

Furthermore, the quality, stability (Benelli et al. 2018), and bioactivity of *Cannabis*-based products depend not only on their cannabinoid content but also on the broader constellation of terpenoids and other specialized metabolites, whose combined profiles define the chemical “fingerprint” of each chemovar and modulate pharmacological responses and receptor–ligand interactions across the diverse genetic backgrounds of the plant (Micalizzi et al. 2021b). Figure 10 summarizes this logic by depicting, in a single scheme, the cannabinoid module (green frame), the monoterpene and sesquiterpene blocks, and the pathways supplying isoprenoid precursors, emphasizing that coordinated variation across all of these axes, rather than changes in the cannabinoid fraction alone, underlies chemical diversity among cultivars.

Genes associated with cannabinoid biosynthesis are generally overexpressed during inflorescence maturation, in parallel with the development of capitate-stalked glandular trichomes, where cannabinoids and terpenoids co-accumulate and where pathway genes form strongly coexpressed modules that segregate chemotypes rich in CBDA (cannabidiolic acid) and in THCA (tetrahydrocannabinolic acid) (Bertoli et al. 2010; Da Porto et al. 2014; Naz et al. 2017; Górski et al. 2016; Zengin et al. 2018; Fiorini et al. 2019; Judžentienė et al. 2023; Pieracci et al. 2021; Smeriglio et al. 2020; Soares et al. 2023; Luca et al. 2024; Benelli et al. 2018; Vuerich et al. 2019; Mazzara et al. 2022; Zheljazkov et al. 2020; Nafis et al. 2019; Rossi et al. 2020). Both male and female inflorescences produce these metabolites, although they are typically more abundant in female flowers, in which cannabidiolic acid or tetrahydrocannabinolic acid predominates according to the underlying chemotype (18,34,89). Integrated gene–metabolite networks therefore indicate that differentiation among chemovars is driven by coordinated transcriptional control of the early steps of biosynthesis and of branch-point enzymes in the cannabinoid and terpenoid pathways, rather than by isolated regulation of cannabinoids alone (Bertoli et al. 2010; Rossi et al. 2020; Ascrizzi et al. 2019). In this context, Fig. 10 can be read as a graphical representation of these regulatory “nodes,” in which branching arrows connect the central precursor module to the structural classes of monoterpenes, sesquiterpenes, and cannabinoids.

Phytocannabinoid biosynthesis in *Cannabis sativa* exemplifies the integration of polyketide and isoprenoid metabolism: the resorcinolic aromatic core is derived from fatty acid–based polyketide steps, whereas the prenyl side chain is supplied by the mevalonate (MVA/MEV) and 2-C-methyl-D-erythritol 4-phosphate (MEP) pathways, as schematically represented in Fig. 10 (Gülck and Møller 2020; Tahir et al. 2021). In the cytosol of secretory disk cells, oxidation of medium-chain fatty acids produces hexanoate, which is activated to hexanoyl-CoA by an acyl-activating enzyme (CsAAE1), a major flux-control step in phytocannabinoid biosynthesis (Stout et al. 2012; Ma et al. 2022). Hexanoyl-CoA and malonyl-CoA are then condensed by tetraketide synthase (TKS) to generate a triketide intermediate that, under the action of olivetolic acid cyclase (OAC), is converted to olivetolic acid, establishing the canonical resorcinolic scaffold (Gagné et al. 2012; Taura and Laroca 2004). In Fig. 10, this polyketide block is positioned immediately below the GPP module, visually highlighting the point at which fatty-acid and isoprenoid fluxes converge.

Subsequent condensation of olivetolic acid with geranyl diphosphate (GPP), an activated monoterpenoid, yields the central intermediate cannabigerolic acid (CBGA), from which THCA, CBDA, CBCA, and other cannabinoid acids are formed by oxidative cyclization catalyzed by specific synthases (Gülck and Møller 2020; Tahir et al. 2021). In parallel, the supply of isoprenoid units is sustained largely by the plastidial MEP pathway, with additional contribution from and cross-talk with the cytosolic MVA pathway, although the precise intercompartmental exchange of intermediates remains only partially elucidated (Oultram et al. 2021; Kaminski et al. 2025). This uncertainty is explicitly indicated in Fig. 10 by the vertical arrow with a question mark that links the blue (MEP) and yellow (MVA) bands, denoting the still poorly understood interchange of intermediates between the two compartments.

Condensation of isopentenyl diphosphate (IPP) and dimethylallyl diphosphate (DMAPP) by geranyl diphosphate synthase (GPPS) produces GPP, which functions both as the immediate precursor of monoterpenes (for example, α-pinene, β-pinene, myrcene, limonene, and terpinolene) and as the prenyl donor for olivetolic acid through specialized prenyltransferases such as CsPT1/CsPT4, thereby generating CBGA (Booth et al. 2017; Rodziewicz et al. 2019). In Fig. 10, this functional duality of GPP is emphasized by its nodal position, connecting the blue monoterpene panel to the large green cannabinoid frame. In contrast, the MVA pathway produces farnesyl diphosphate (FPP), the key precursor of sesquiterpenes such as β-caryophyllene and α-humulene, which are abundant constituents of most *Cannabis* essential oils (Booth et al. 2017; Rodziewicz et al. 2019). These sesquiterpenes are highlighted in the upper yellow panel of Fig. 10, thereby visualizing the separate yet interconnected families of GPP-derived monoterpenes and FPP-derived sesquiterpenes. Thus, GPP occupies a nodal position at the interface between monoterpene and cannabinoid biosynthesis, whereas FPP supports sesquiterpene formation, establishing a biosynthetic network in which cannabinoids and terpenoids simultaneously compete and cooperate for a shared “pool” of isoprenoid precursors; transcriptomic and gene-network analyses corroborate this interdependence by revealing co-expression of terpene synthases (TPS), prenyltransferases, and cannabinoid synthases in floral trichomes (Booth et al. 2017, 2019; Desaulniers Brousseau et al. 2021).

This integration is profoundly shaped by subcellular compartmentalization. Microscopy, proteomic, and systems biology studies indicate that phytocannabinoid biosynthesis is partitioned among three main domains in capitate-stalked glandular trichomes: the cytosol, plastids, and the apoplastic or subcuticular cavity (Tanney et al. 2021; Arif et al. 2021). The polyketide phase (formation of hexanoyl-CoA, TKS, and OAC) occurs in the cytosol of secretory cells, whereas prenylation of olivetolic acid, supplied with GPP from the MEP pathway, is associated with plastid membranes; the final oxidative cyclization of CBGA to THCA, CBDA, or CBCA is catalyzed by secreted flavoproteins (THCAS, CBDAS, CBCAS) in the apoplastic lumen, with the resulting cannabinoid acids accumulating in the subcuticular storage cavity (Gülck and Møller 2020; Sirikantaramas et al. 2005; Xie et al. 2023). Terpenoid biosynthesis follows a similar compartmental logic: GPP-derived monoterpenes are synthesized by plastidial TPSs, whereas many sesquiterpenes are produced by cytosolic TPSs using FPP from the MVA pathway; all of these volatile products accumulate in the same resin-filled cavity (Booth et al. 2019; Tanney et al. 2021). Although Fig. 10 depicts these routes in simplified form, the spatial juxtaposition of the monoterpene, sesquiterpene, and cannabinoid modules alludes to the functional colocalization of these metabolites in glandular trichomes.

Comparative proteomes of trichome heads and stalks, as well as of whole floral tissue, reveal a marked enrichment of MEP-pathway enzymes, TPSs, CsPTs, and cannabinoid synthases in trichome heads, reinforcing their designation as highly polarized “supercells” specialized in the biosynthesis and storage of secondary metabolites (Conneely et al. 2021; Livingston et al. 2022). This spatial organization creates metabolic microdomains in which the physical proximity of enzymes promotes channeling of intermediates, reduces diffusion of reactive species, and limits exposure of CBGA and THCA to the cytosol, thereby mitigating cellular cytotoxicity (Deguchi et al. 2020, 2022). The productivity and chemical quality of *Cannabis* products emerge directly from this integrated architecture. The distribution of carbon flux between the MEP/MVA pathways and the polyketide pathway determines the size of the precursor “pools” available for CBGA formation and for the production of volatile terpenes. Systems biology models and gene expression studies indicate that, in trichome-rich floral tissues, the MEP pathway supplies most of the IPP/DMAPP, such that limitations in this pathway lead to coupled reductions in both monoterpenes and cannabinoids (Tanney et al. 2021; Mediavilla and Steinemann 1997). Conversely, higher GPPS or CsPT activity tends to divert GPP from monoterpene synthesis toward CBGA formation, thereby rebalancing the relative contributions of “aroma” (terpenoids) and “potency” (cannabinoids) in the resin (Booth et al. 2017; Wiles et al. 2022). This balance between “aroma” and “potency” is schematically suggested in Fig. 10 by the central placement of GPP between the monoterpene block and the cannabinoid module.

In parallel, the development and density of capitate-stalked trichomes are primary determinants of metabolite yield per unit of cultivated area; recent reviews describe positive correlations between trichome density, THCA/CBDA levels, and terpene concentrations in female flowers, under the control of complex hormonal networks (jasmonates, salicylic acid, gibberellins) and specific transcription factors (Tanney et al. 2021; Xie et al. 2023; Dimopoulos et al. 2025). Agronomic or breeding strategies that enhance trichome formation and coactivate genes of the MEP/MVA pathways, TPSs, and cannabinoid synthases therefore generate richer and more stable resins. Compartmentalization also governs the stability and integrity of metabolites within the plant matrix. Storage of cannabinoid acids and terpenes in subcuticular cavities reduces their exposure to enzymatic or non-enzymatic oxidation, helping preserve chemical profiles during floral development and the initial postharvest phase (Tanney et al. 2021; Arif et al. 2021). However, variation in cuticle thickness, secretion dynamics, and responses to abiotic stress (for example, high light or water deficit) can alter the rate of terpene volatilization and of cannabinoid-acid decarboxylation or degradation, thereby modulating the final aromatic bouquet and pharmacological potency (Sommano et al. 2020; Contreras-Avilés et al. 2024).

Metabolomic and chemometric analyses demonstrate that the combined signatures of cannabinoids and terpenes define chemovars and correlate strongly with perceived quality attributes, including organoleptic profile, pharmacological effects, and market value (Kaminski et al. 2025; Booth et al. 2020). Consequently, a detailed understanding of integrated biosynthesis and compartmentalization, as summarized schematically in Fig. 10, which positions GPP as a central hub connecting monoterpenes, sesquiterpenes, and cannabinoids, is a prerequisite for the rational design of cultivars, cultivation protocols, and metabolic engineering approaches aimed at maximizing productivity and finely tuning the chemical quality of *C. sativa* products for medicinal and industrial applications.

### Characteristics of the data collected on *Cannabis* volatile oils: time trend analysis

The cultivation of *Cannabis* spp. for VO extraction has shown significant trends in genotype preferences and geographical distribution over the past few decades (Table 1). Among the most cultivated genotypes, Carmagnola, Futura *75*, and Kompolti stand out due to their adaptability and high yield potential, particularly in Italy, which emerges as a key region for *Cannabis* diversity and research. In recent years, the consistent use of Felina *32* and Carmagnola selezionata further highlights the importance of optimized cultivars for VO production.

Over time, research activity has notably increased, with publication peaks observed between 2019 and 2021, reflecting growing scientific interest in *Cannabis* VOs driven by regulatory changes, expanding therapeutic applications, and the increasing use *Cannabis* seeds in the food industry (Baldini et al. 2018). Additionally, regional specialization—such as the use of Kompolti hybrids in Switzerland and indigenous landraces in Pakistan and Morocco—illustrates how ecological and cultural factors influence genotype selection. These findings underscore the importance of selecting robust, high-yield genotypes and leveraging geographical conditions to maximize EO quality and production.

The Table 1 demonstrate the correlation between different regions and the number of genotypes cultivated for *Cannabis* VOs. The regions with the highest diversity of genotypes include Udine, Ragusa, and Rovigo, in Italy, highlighting Italy's significant role in the cultivation of diverse *Cannabis* genotypes. Other prominent regions, such as Zurich, Switzerland, and Amstetten, Austria, also demonstrate a substantial number of genotypes, emphasizing the central role of these regions in *Cannabis* research and production. This visualization reinforces the geographical trends and the specialization of specific genotypes in various regions.

### Chemical stability of cannabinoids in the post-harvest process

From cultivation to commercialization, *Cannabis* undergoes several processing phases, during which the degradation of key cannabinoids can occur (Addo et al. 2021; Rathore and Mathur 2019; Nursuhaili et al. 2019). The processing of *Cannabis* inflorescences significantly influences cannabinoid degradation, a factor that should not be underestimated. In intact glandular trichomes, cannabinoids are well-protected against oxidative degradation due to their encapsulation within these specialized structures. A similar principle applies to the preservation of terpenoids (Veit 2023). Therefore, developing optimized processing technologies tailored to different production scales is crucial. For example, cutting and drying technologies are essential for maintaining the chemical integrity of cannabinoids, as these processes heavily influence the quality and stability of the final product (Kwaśnica et al. 2020a; Addo et al. 2021; Kotan et al. 2007).

In the *Cannabis* industry, trimming is a critical operation that directly impacts the production of high-quality products. During harvest, many companies remove the sugar leaves (bract leaves) surrounding the inflorescence to enhance both the visual appeal and cannabinoid concentration of the final product. Some companies also perform threshing and cutting after the drying phase. However, it is essential to optimize these processes to prevent the loss of key chemical constituents and to preserve the integrity of the trichomes, the glandular structures that produce the plant's main cannabinoids and terpenes, thereby maintaining the therapeutic and commercial value of the product (Chandra et al. 2020, 2017; Sparks 2025; Lorensen et al. 2023). The interaction between cannabinoids and terpenes can also significantly enhance therapeutic outcomes. For instance, the monoterpene α-pinene has been identified as an acetylcholinesterase inhibitor, potentially counteracting the psychoactive effects of Δ^9^-THC (Kennedy et al. 2011). Meanwhile, β-caryophyllene, a commonly occurring sesquiterpene, interacts with cannabinoid receptor type 2 (CB2) to exert anti-inflammatory effects (Micalizzi et al. 2021b). These findings emphasize the dual role of terpenes as both therapeutic agents and essential components in the formulation of more effective *C. sativa*-based medications.

Another critical factor in *Cannabis* post-harvest processing is reducing the moisture content of the inflorescence, which tends to have high humidity levels. Moisture reduction is essential for safe storage and maintaining product quality. Water activity (wa), closely linked to moisture content, must be lowered below critical levels to prevent contamination and ensure product safety. Safety concerns must be addressed, particularly the allergenic properties of certain terpenes, which are especially relevant in cosmetics, where the European Union mandates clear labeling of these substances (CosIng Database). Additionally, terpenes could play a crucial role in resolving taxonomic classification challenges arising from the widespread genetic modification of *C. sativa* cultivars. These modifications, aimed at producing higher concentrations of secondary metabolites like Δ^9^-THC or CBD, have been central to the illicit market for years, particularly when *C. sativa* trade was illegal (Chandra et al. 2020). This raises an important question: what are the barriers to adopting the most effective processing technologies? A recent review of *Cannabis* drying methods emphasized the need for further research into optimal drying parameters and models. While drying remains the most common method for preserving medicinal plants, it is resource*-*intensive, requiring substantial energy and financial investment. These challenges significantly impact the production process (Chandra et al. 2017; Kwaśnica et al. 2023).

Efficient and sustainable drying technologies are still evolving, and various strategies, including pre*-*treatments, are being developed to enhance the drying process. Drying methods using convection, conduction, radiation, or their combinations are well-established in both the medicinal plant and food industries. These approaches aim to optimize drying time while preserving the chemical integrity of the final product. (Chandra et al. 2020) reviewed various pre*-*treatment techniques for *Cannabis*, including chemical and physical methods, and compared them to advanced technologies like microwave*-*assisted drying. This method shows significant promise by increasing operational speed, improving product quality, enhancing energy efficiency, and reducing costs. Studies, such as (Fiorini et al. 2019), have demonstrated that the microwave*-*assisted drying can produce high-quality, safe, and cost-effective products (Chandra et al. 2020) also introduced the novel application of cold plasma technology, a non-thermal technique used in the medicinal plant and food industries. Cold plasma has been successfully applied to products like wolfberries, saffron, and lemon verbena. This technology offers several advantages, including the ability to eradicate microorganisms, inactivate enzymatic activity, and improve product safety without thermal degradation.

The *Cannabis* drying process still relies predominantly on traditional, slow, low-temperature methods, typically taking 5 to 6 days. Currently, no standardized model exists to accurately predict the drying endpoint or total duration, making the post-harvest process more of an art than a science. To address this gap, several modern drying techniques have been proposed, including microwave*-*assisted convective drying, freeze*-*drying, vacuum drying, microwave*-*assisted freeze*-*drying, intermittent drying, belt drying, radiofrequency drying, and electrohydrodynamic drying. These advanced methods hold promise in optimizing the drying process by reducing drying time while preserving the chemical integrity of key constituents.

Psychrometry, the study of the thermodynamic properties of air, is crucial in the *Cannabis* industry, particularly for quality control, storage, and packaging. Key parameters such as vapor pressure, relative humidity, moisture content, and water activity (wa) are essential for effective drying. These variables must be thoroughly investigated and optimized for both drying and storage under varying environmental conditions to ensure product stability and safety (Dincer and Rosen 2021; Balmer 2011; Al Ubeed et al. 2022; Chen et al. 2021). Determining the moisture equilibrium of medicinal plants, including *Cannabis*, is critical for establishing drying parameters, storage protocols, and marketing strategies, such as the direct sale of dried *Cannabis*. Additionally, the optimal range of wa, which differs from moisture content, must be carefully defined and controlled (Unger et al. 2014; Boyar 2021).

The *Cannabis* inflorescence contains a high initial moisture content, ranging from 75–78%, making it highly perishable before the drying process. Inadequate control and prediction methods for *Cannabis* drying and storage can lead to mold formation, compromising both quality and safety. Factors such as water permeability and the rigidity of packaging materials are critical for maintaining consistent moisture levels and preventing mechanical damage during storage and transport (McKeen 2017). A comprehensive approach that considers diffusion coefficients, sorption isotherms, and packaging permeability can help producers make decisions to enhance production efficiency and product quality (Chandra et al. 2020).

Common packaging materials in the *Cannabis* industry include Mylar bags, polyethylene terephthalate (PET), metal boxes, tubes, and glass jars (McKeen 2017). Additionally, storage conditions, particularly temperature and light exposure, are significant factors contributing to the degradation of *Cannabis* and its cannabinoids. Elevated temperatures and prolonged light exposure accelerate the degradation process, reducing both cannabinoid concentration and overall product quality (Trofin et al. 2012a). Therefore, temperature control during storage is essential to preserve the moisture content and maintain the cannabinoid potency in dried *Cannabis* inflorescences (Trofin et al. 2012a, 2012b). The challenges associated with *Cannabis* post-harvest processing share similarities with those in other industries, such as medicinal plants and perishable food products. By adopting technological strategies from these sectors, significant advancements in *Cannabis* processing can be achieved. The incorporation of innovative technologies could improve the sustainability, scalability, and economic viability of *Cannabis* production, enhancing both its therapeutic value and commercial potential.

Sterilization is critical in the medical *Cannabis* industry to ensure safety from microorganisms. In Israel, the safe limit for total mold and yeast (TMY) colony-forming units (CFUs) in medicinal *Cannabis* inflorescences is 2000 TMY CFUs/g of dried inflorescence. However, traditional sterilization techniques, such as autoclaving, may compromise the chemical integrity of the product by degrading key constituents like terpenes and cannabinoids, which are sensitive to heat and UV radiation. To mitigate these effects, the industry has explored alternative sterilization methods, including ethylene oxide (EtO), electron beam (*E-*beam) irradiation, gamma irradiation, and steam sterilization. Each of these methods has distinct advantages and drawbacks in terms of their impact on the chemical composition of *Cannabis* inflorescences. For example, vapor sterilization, while effective, can degrade terpenes and cannabinoids depending on exposure time and temperature, as shown a previous study (Jerushalmi et al. 2021).

Further research by Dhillon et al. (2022) compared the effectiveness of four decontamination techniques, gamma irradiation, *E-*beam irradiation, steam sterilization, and hydrogen peroxide vapor treatment, and assessed their effects on cannabinoid content. While all techniques were effective in reducing microbial contamination, the study revealed that each method impacted the cannabinoid profile of the *Cannabis* samples to varying degrees. Given these findings, future research should focus on optimizing sterilization techniques that preserve the stability of both major and minor cannabinoids, as well as terpenes, to maintain the therapeutic efficacy and quality of medical *Cannabis*.

The degradation of cannabinoids and terpenoids is primarily driven by heat, oxidation, and prolonged exposure to solvents solutions. Temperature and exposure time are key factors influencing the stability of these compounds. For example, prolonged exposure to high temperatures during extraction can convert Δ^9^-THC to cannabinol (CBN), which alters the chemical composition and can also reduce both the efficacy and quality of the final product. Oxidation, particularly in terpenoids, can leads to the degradation of key compounds responsible for flavor and aroma, significantly affecting the sensory and therapeutic properties of the product. Given these challenges, optimizing extraction techniques and processing conditions is critical to minimize degradation and preserve the pharmacological potency of cannabinoids and terpenoids. Variables such as solvent choice, temperature, pressure, and exposure time must be carefully controlled to maximize product quality and efficacy.

The selection of extraction procedures and subsequent production steps, such as winterization, decarboxylation, and evaporation, can significantly influence both cannabinoid content and the chemical stability of the product (Trofin et al. 2012b; Kessler and Kalske 2018). This is a critical consideration not only for large*-*scale producers but also for small and medium-sized enterprises involved in *Cannabis* production. A study by Calvi et al. (2018) examined the stability of cannabinoids in medicinal oils prepared using three different extraction methods. The results highlighted the importance of the chosen methodology, showing that cannabinoid stability is directly related to the preparation technique. These findings underscore the need to standardizing extraction and processing methods to ensure consistency in medicinal *Cannabis* oil production across the industry.

The chemistry of cannabinoids is complex, encompassing both acidic and neutral (decarboxylated) cannabinoids. Each of these compounds features a resorcinolic nucleus and a side chain that varies in length (5 or 3 carbon atoms). The bicyclic region, as well as the aliphatic chain, is particularly sensitive to oxidation. Notably, the single double bond at the Δ9- position exhibits poor oxidative stability during the storage of dried plant material. Furthermore, cannabinoid decarboxylation is a spontaneous and non-enzymatic process that strongly depends on temperature. Studies also suggest that microbial degradation may lead to side*-*chain oxidation (Thomas and Kayser 2022).

To minimize the degradation of cannabinoids and terpenoids, extraction techniques and processing conditions must be carefully optimized. Key factors such as solvent choice, temperature, pressure, and exposure time should be meticulously controlled to maximize product quality and effectiveness. In the *Cannabis* industry, common extraction methods include solvent extraction, cold-press extraction, supercritical CO₂ extraction, microwave*-*assisted extraction, and ultrasound-assisted extraction. Among these, solvent and supercritical CO₂ extraction are the most widely employed. However, developing large*-*scale extraction protocols that are both efficient and sustainable remains a significant challenge.

**Fig. 11:**
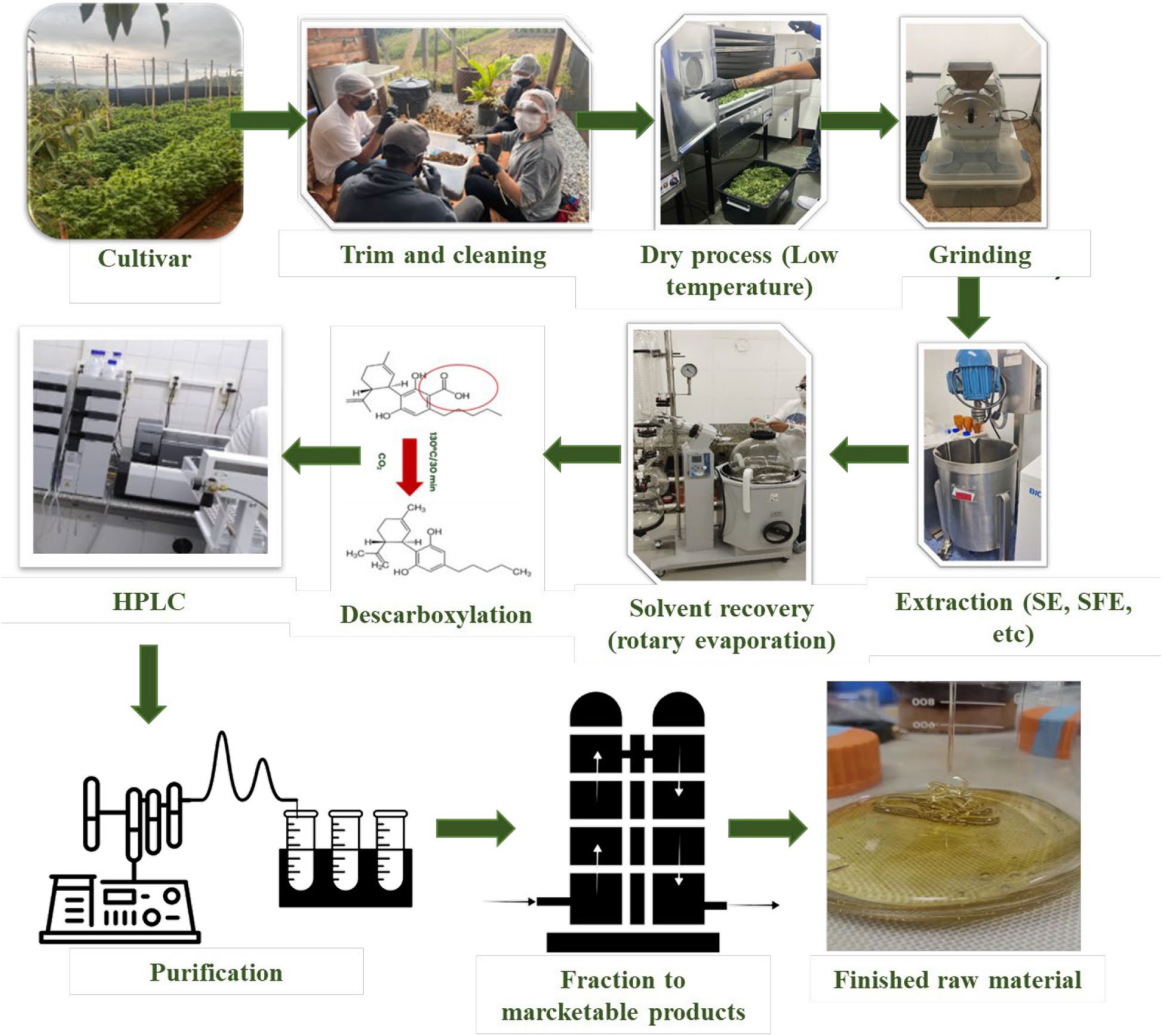
Flowchart of the *Cannabis* processing steps for cannabinoid extraction. Legend: HPLC = High Performance Liquid Chromatography, SE = Solvent Extraction, SFE = Supercritical Fluid Extraction

Based on the understanding of critical post-harvest processes (Fig. 11), several key questions emerge that could further advance the production of high-quality and safe *Cannabis* products. For example, can existing extraction methods be combined with novel techniques not yet utilized in medicinal *Cannabis* production? Recent literature discusses green harvesting for the extraction of secondary metabolites (Torres-Ortiz et al. 2024), suggesting that pre*-*treatment methodologies could enhance the extraction process from. While studies have addressed these issues, consensus remains elusive, particularly regarding the scalability of bench-top production to medium- or large*-*scale operations (see (Fathordoobady et al. 2019; Leghissa 2018)).

Research by Valizadehderakhshan et al. (2021) highlights various extraction method and their potential uses in pre*-*treatment techniques. A key question arises: how do these pre*-*treatments impact mass transfer efficiency? Techniques such as high-pressure homogenization, pressing, ultrasonication, microwave assistance, acid lysis, enzymatic treatments, and osmotic shock have been explored. More conventional methods, including grinding, tearing, or gently crushing *Cannabis* inflorescences, can reduce solvent requirements. However, excessive grinding may extract undesirable compounds like chlorophyll and waxes from the internal tissue matrix, complicating purification and potentially yielding non-pharmaceutical grade products (Al Ubeed et al. 2022).

Microwave*-*assisted extraction utilizes microwaves to penetrate tissue and generate heat, facilitating biomass disintegration. While this thermal energy aids extraction, it also risks cannabinoid degradation. Further research is needed to fully understand these effects. Additionally, this process can promote decarboxylation, which is beneficial when neutral cannabinoids are the desired outcome (Fiorini et al. 2019).

The post-extraction phase, particularly winterization, remains an under-researched area. This process, which involves removing waxes and pigments at low temperatures, can result in the loss of some terpenes and cannabinoids (Valizadehderakhshan et al. 2021; Marzorati et al. 2020; King 2019). To better understand the underlying physicochemical processes, it is crucial to evaluate winterization’s impact on cannabinoid chemical stability at low temperatures. Key questions include whether extreme cold (- 40 °C) and heat alter the thermodynamic and kinetic behavior of degradation reactions, as well as the optimal temperature and duration for winterization. Addressing these complexities of post-harvest processing requires a multidisciplinary approach (Baldino et al. 2020).

Despite advancements in extraction techniques, critical questions remain. How does water activity influence the chemical and structural stability of *Cannabis* extracts? What is the optimal water activity level for *Cannabis* at different temperatures? These parameters are essential for developing software and digital tools to optimize *Cannabis* production processes. Additionally, how can sensor technology and digital integration support informed post-harvest management decisions?

Extraction methods significantly impact production costs, yields, and product quality. While research has focused on cost reduction, efficiency, sustainability, and byproduct utilization, a deeper understanding of chemical transformations and artifact formation during extraction is crucial. Physicochemical and thermodynamic interactions between extraction methods and instrumentation play a key role in these processes.

Optimizing *Cannabis* production requires a comprehensive understanding of post-harvest methodologies. These methods influence yield, purity, and the potential for compound alteration due to factors like temperature, pressure, and water activity. Addressing these challenges is essential for refining post-harvest protocols and ensuring the production of high-quality, (Good Manufactory Practice) GMP-compliant *Cannabis* products.

### Chemical stability of Δ^9^-tetrahydrocarboncannabinol (Δ^9^-THC) and cannabidiol (CBD) for final product: Storage and packaging

The chemical stability of cannabinoids is a critical aspect of the packaging and storage of *Cannabis*-derived products (MacLaughlin and MacDonald 2024). Exposure to adverse conditions, such as light, air, temperature, and humidity, trigger to degradation processes, including decarboxylation, isomerization, photoirradiation, and oxidation. These reactions may produce unwanted metabolites, compromising both the quality and safety of the final product. While most studies have focused on Δ^9^-THC and CBD, available data remain limited and often context-specific. A key unresolved question is: What are the primary thermal degradation products of cannabinoids? Until this gap is adequately addressed through characterization studies and toxicological assessments, careful consideration of storage, transportation, and other factors remains essential.

Understanding how processing and storage affect cannabinoid stability is crucial, especially for lesser-known compounds with uncertain psychotropic and biological activity (Kanabus et al. 2021) Stability studies play a key role in evaluating the impact of physical, chemical, and environmental factors on cannabinoid preservation, ensuring compliance with established quality standards. These studies also help determine optimal storage and packaging conditions to minimize degradation and extend shelf life. Improper transport and storage can accelerate cannabinoid degradation through oxidation, isomerization, dehydration, polymerization, and thermal rearrangement. For example, Δ^9^-THC can oxidize into cannabinol (CBN) when exposed to light, air, or high temperatures. Similarly, CBD can undergo chemical cyclization, converting into Δ^9^-THC, which may then oxidize into CBN (Jaidee et al. 2022).

The oxidative degradation of Δ^9^-THC can be mitigated, or at least slowed, by protecting it from atmospheric oxygen, with suitable packaging playing a crucial role. Storing and transporting *Cannabis* in oxygen-free conditions helps preserve the integrity of the product (Veit 2023). Hermetic sealing in modified atmosphere packaging (MAP), which typically includes high-barrier gas structures with low oxygen transmission rates (OTR), has proven effective in the food industry. However, it remains to be seen whether this technology can be equally effective for *Cannabis-*based products (Holste and Schoeber 2019).

MacLaughlin et al. (MacLaughlin and MacDonald 2024) explored the preservation of plant cuticles, whose primary macromolecular component is cutin, a polyester of covalently linked C16 and C18 hydroxylated fatty acids. In their study, they examined the oxidation of cuticles by reactive oxygen species (ROS) present in atmospheric air, which leads to the degradation of the cell wall and accelerates the breakdown of chemical constituents in trichomes. MacLaughlin et al.’s (MacLaughlin and MacDonald 2024) findings highlight the need for further research to confirm the effectiveness of MAP in preserving cannabinoids, as there is currently insufficient evidence supporting its efficacy in this context. This presents a critical point for future study and validation.

Oxidative degradation is thermodynamically controlled and can be slowed by storing products at low temperatures. Lower temperatures also inhibit microbial growth, reducing the risk of secondary contamination of dried *Cannabis* inflorescences. However, concerns remain regarding storage at freezing or subfreezing temperatures. Glandular trichomes, responsible for cannabinoid accumulation, may be damaged at these low temperatures, exposing cannabinoids to oxygen and potentially accelerating Δ9-THC degradation. Similarly, excessively dry storage conditions can also lead to trichome brittleness, making cannabinoids more susceptible to degradation. Optimal storage conditions for *Cannabis* inflorescences are typically between 55 and 62% relative humidity, preventing both desiccation and the breakdown of trichome structures*.* In contrast, a study of *Cannabis* resin stored in freezers found that stored in plastic bags showed no degradation of Δ^9^-THC, CBD, or CBN after 4 years (Grafström et al. 2019).

In the food industry, research on innovative packaging technologies consistently explores new methods and materials to extend product shelf life. Oxidative degradation remains a major challenge in food preservation, and solutions such as oxygen absorbers have been shown to reduce oxygen concentration to as low as 100 ppm. Applying similar packaging technologies to the *Cannabis* industry could help maintain cannabinoid chemical stability, ensuring product quality throughout the supply chain.

A recent study also highlighted the potential of vapor-phase terpenes in reducing oxidative degradation of cannabinoids during *Cannabis* inflorescence storage. This research examined the antioxidant properties of exogenous terpenes and their role in cannabinoid stabilization. After 127 days of storage, inflorescences stored with vapor-phase terpenes exhibited a 47.4% reduction in Δ^9^-THC degradation compared to samples stored without terpenes (MacLaughlin and MacDonald 2024).

These examples highlight the critical need for comprehensive studies on cannabinoid stability across various *Cannabis*-derived products, such including medicinal oils, extracts, dried inflorescences, and edibles, where understanding stability within different food matrices is essential. Additionally, it is crucial to investigate how extrinsic factors, such as transportation logistics, packaging materials, and storage conditions, affect cannabinoid degradation. For example, the German Pharmacopoeia (2020) specifies that the concentration of cannabinol (CBN) in *Cannabis* inflorescences must not exceed 1.0%, as CBN serves as an indirect indicator of product quality and reflects the effectiveness of processing, packaging, and storage conditions.

### Limitations and future perspectives

**Fig. 12:**
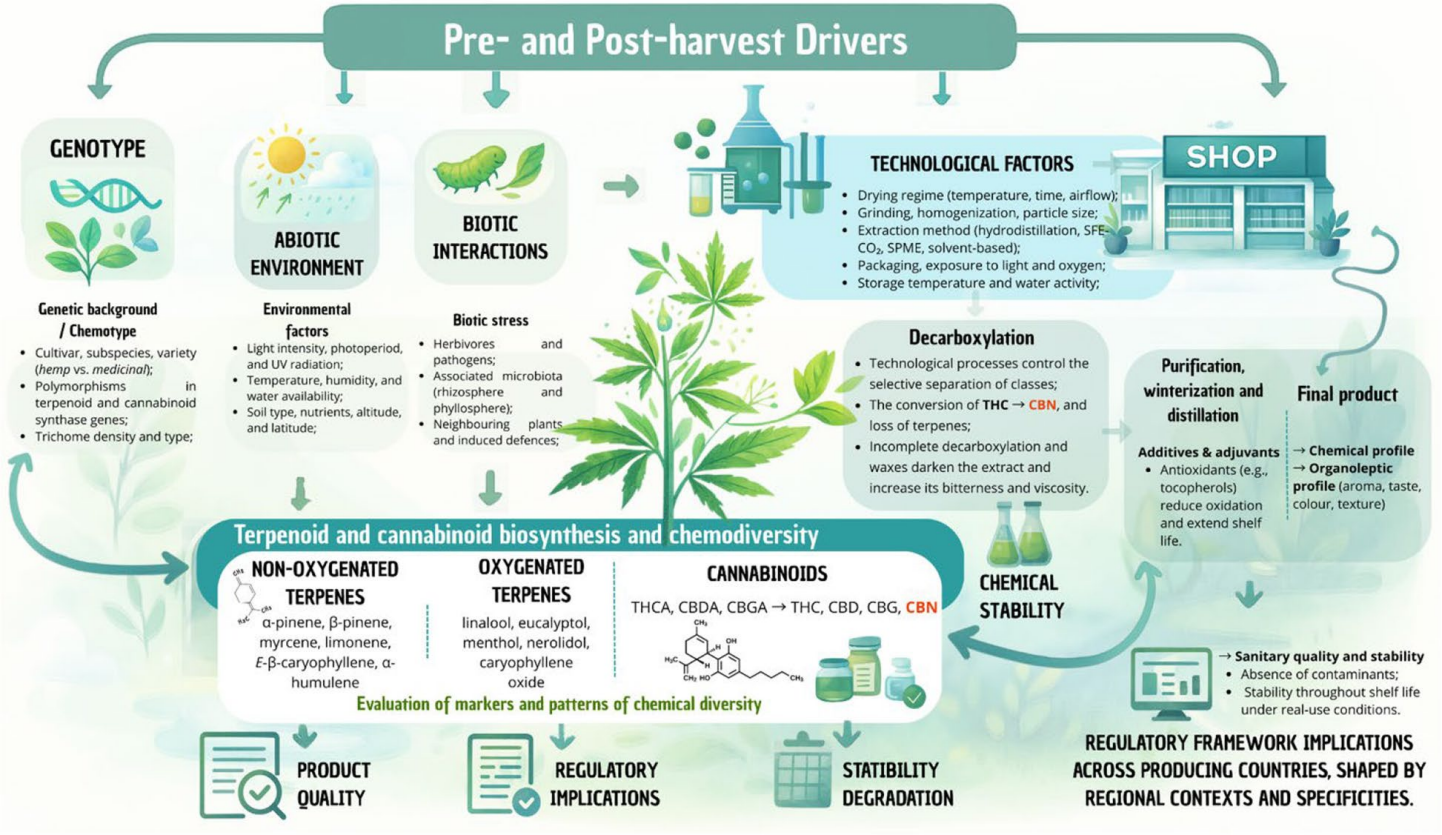
Conceptual diagram integrating the main pre- and post-harvest determinants in the *Cannabis sativa* “crop-to-shop” chain

Figure 12 provides an overview of the major pre- and post-harvest drivers that shape chemical diversity, stability, and regulatory outcomes in plant-derived products. By integrating genetic, biotic and abiotic effects, and technological factors, the model underscores the multifactorial nature of terpene and cannabinoid biosynthesis. Yet, despite this comprehensive perspective, key uncertainties remain regarding the relative influence of each driver, their interactions, and the consistency of chemical traits under real production conditions. Addressing these gaps will be essential for improving predictive capacity, enhancing product quality and safety, and supporting more coherent regulatory frameworks.

### Limitations

The reviewed studies revealed several methodological gaps that impact the robustness of the available evidence regarding the chemical stability and chemodiversity of *C. sativa*. One major issue is the lack of harmonization in analytical methodologies for volatile and non-volatile compounds, leading to discrepancies in reported data. Furthermore, the predominance of studies conducted in certain regions, such as Europe, and the scarcity of research in Latin America and Africa introduce a geographical bias that limits the global applicability of the findings.

Another critical gap is the lack of investigations into the influence of ecological and evolutionary factors on the chemical expression of *C. sativa*. Most studies have focused on pharmacological and industrial aspects, leaving significant gaps in understanding the species’ chemical ecology. Additionally, few studies have explored the relationship between environmental conditions and the plant’s phenotypic plasticity, a key determinant of the production of secondary metabolites and, consequently, of the quality of final products.

Moreover, most research has primarily investigated major compounds, such as Δ^9^-THC and CBD, while secondary metabolites like CBC and THCV have received limited attention. For the volatile fraction specifically, cannabinoids present in the EO were not consistently quantified, and, when reported, their levels differed markedly among samples, which hinders direct comparison across chemotypes. In our dataset, all essential oil samples that reported cannabinoid contents exhibited distinct and non-standardized levels, such that the integrative assessment of cannabinoids and terpenoids in the same volatile matrix was constrained. As our review was designed around terpenoid profiles, this inconsistency in cannabinoid quantification within VOs represents an additional limitation to fully capturing the entourage effect between these two classes of metabolites. Taken together, this gap, taken together, constrains the understanding of the therapeutic and commercial potential of these compounds and their interactions in the entourage effect.

Our study sought to explore the chemical stability and chemodiversity of *C. sativa* throughout the production chain, from cultivation to commercialization. However, several limitations must be acknowledged. First, the genetic and phenotypic variability of *Cannabis*, combined with environmental and cultivation conditions, presents significant challenges for standardizing chemical composition. The heterogeneity of analyzed samples and the diverse methodologies employed in the reviewed studies may have influenced the comparability of results, restricting the generalization of conclusions. In addition, incomplete or inconsistent reporting of key metadata (for example, genotype, developmental stage, post-harvest history, and storage conditions) in several primary studies limits the depth of possible subgroup analyses and the reconstruction of causal links between processing steps and chemical outcomes.

Additionally, despite the use of advanced statistical tools, such as principal component analysis (PCA) and hierarchical clustering analysis (HCA), the limited data available on intra- and inter-regional variations hinder the identification of consistent chemical composition patterns. This study also faced challenges related to the lack of standardization in extraction and post-harvest processing techniques, as well as the absence of uniform methodologies for assessing the chemical stability of compounds over time. Another critical limitation is the scarcity of studies examining the relationship between the chemical stability of phytocannabinoids and terpenoids and their degradation processes during storage and processing, highlighting the need for further investigation. Finally, as a structured scoping review based exclusively on published data, our synthesis is inherently descriptive and may be affected by publication bias and the underrepresentation of negative or inconclusive results, reinforcing the need for controlled, longitudinal experiments to validate the patterns proposed here.

### Future research direction

*Perspective 1:* Future studies should adopt integrated approaches combining natural product chemistry, metabolomics, genetics, chemical ecology, and systems biology to better understand the mechanisms governing the production of specialized metabolites in *C. sativa*.

*Perspective 2:* Investments in standardized quality control protocols throughout the production chain are essential. The development of robust analytical methods, including mass spectrometry and nuclear magnetic resonance (NMR) techniques, will enable more precise monitoring of *Cannabis* chemical composition.

*Perspective 3:* Research on the effects of environmental factors, such as temperature, UV radiation, humidity, and biotic and abiotic stresses, on the stability and chemical variation of bioactive compounds is crucial for optimizing cultivation and ensuring production consistency. This is particularly relevant for regions in the Americas (North, Central, and South), Africa, and Oceania, where spatiotemporal patterns differ significantly.

*Perspective 4:* Studies on minor compounds, including rare cannabinoids and underexplored terpenoids, could reveal novel pharmacological properties and expand the therapeutic and industrial applications of *C. sativa*. Despite the increasing volume of studies, there is a need for more robust analytical techniques to isolate, purify, and identify new bioactive compounds, particularly in ecosystems where this species is encountering novel environmental conditions for the first time in its evolutionary history.

*Perspective 5:* Research into the application of gene*-*editing techniques and marker-assisted selection could facilitate the development of cultivars with optimized chemical profiles, enabling the production of specific chemotypes for diverse medical and industrial applications.

*Perspective 6:* Investigations that evaluate the chemical stability of *Cannabis* and its derivatives over time, considering different storage and processing methods, are necessary to ensure product quality for end users.

*Perspective 7:* The development of more efficient and sustainable extraction methods for cannabinoids and terpenoids, such as ultrasound-assisted extraction and supercritical fluid extraction, could enhance the yield and purity of extracts.

*Perspective 8:* Studies on the impact of regulatory policies on the quality and accessibility of medicinal *Cannabis* products are essential to guide the development of more effective and inclusive public policies.

*Perspective 9*: Future research should systematically quantify phytocannabinoids in essential oils and other volatile fractions, using harmonized and validated analytical protocols. Integrating cannabinoid and terpenoid datasets obtained from the same matrices and along the production pipeline (flowers, resins, essential oils, and finished products) would enable a more precise assessment of how volatile cannabinoids contribute to pharmacological, sensory, and stability profiles, and how processing steps modulate these interactions within the entourage effect. Such approaches should also prioritize geographically diverse and underrepresented regions, particularly in the Global South, to better capture the full spectrum of chemodiversity and production realities.

Additional research is essential to move toward the standardization of *Cannabis* compounds as modern therapeutics. Achieving this goal requires substantial corporate and public investment to promote integrative approaches across diverse fields, such as pharmacology, molecular biology, botany, agronomy, ecology, and chemistry. This multidisciplinary collaboration is crucial to overcoming the fragmented nature of current research and development. Although this integrated approach is not new—originally emphasized by Dr. Otto Richard Gottlieb for studies on medicinal plants (Gottlieb et al. 1998), it now needs to be practically implemented.

The processes involved in the production and extraction of *Cannabis* compounds, including drying and decarboxylation, significantly impact their pharmacological properties, sensory characteristics, and overall yield (Lewis-Bakker et al. 2019). Therefore, it is crucial to establish clear quality parameters, as these are essential for ensuring that pharmaceutical products meet established bioactivity standards (Kessler and Kalske 2018). However, this becomes challenging when using crude *Cannabis* extracts, as variations between chemotypes and plant matrices often correlate with different therapeutic applications (Gorelick and Bernstein 2017; Bridgeman and Abazia 2017; Brodie and Ben-Menachem 2018).

In addition, chemical stability, understood as the maintenance of consistent chemical profile across generations, phenotypic expression, and through different stages of processing, is critical to achieving quality. This *Cannabis* quality depends on various factors, including physicochemical, genetic, metabolic, and proteomic aspects, as well as applied agronomic practices applied and environmental influences on different cultivars. Research indicates that the levels of cannabinoids and terpenoid phenolics can be sensitive to degradation, influenced by factors such as environmental conditions, cultivation methods, and post-harvest processes (Gouvêa-Silva et al. 2023; Pavlovic et al. 2018; Caprioglio et al. 2020; Vacek et al. 2021).

To ensure effective quality control, it is necessary to employ classical phytochemical analyses alongside modern analytical techniques, such as omics sciences (Cerrato et al. 2021; Andre et al. 2016; Aliferis and Bernard-Perron 2020). While these methods have demonstrated positive outcomes (Benchoula et al. 2022; Mattoli et al. 2023), additional measures are required to enhance the quality and standardization of *Cannabis* products, from cultivation to commercial distribution (Hinckley et al. 2022; Bautista et al. 2021). It is also essential to address how both intrinsic factors (*e.g*., somatic mutations, epigenetic variations) and extrinsic factors (*e.g*., environmental stressors, cultivation practices, extraction procedures) affect the safety, quality, and efficacy of the final products (Kwaśnica et al. 2020a; Ramos et al. 2020; Costa-Oliveira et al. 2021). Moreover, while the impact of biotic and abiotic stress on the production of secondary metabolites is well understood for many plants (Jan et al. 2021), more research is needed on *Cannabis* varieties to fully understand these dynamics (but see (Liu et al. 2021; El-Mernissi et al., 2024; Gulluni et al., 2018; Joy et al., 2025; Kwasnica et al., 2020b; Tabis et al., 2024; Janta and Vimolmangkang 2024; Herwig et al. 2025).

Addressing these gaps and challenges will contribute to advancing knowledge on *C. sativa*, promoting its safe, effective, and sustainable use across various sectors. It is necessary to take a leap in *Cannabis* research, moving beyond an analysis restricted to a single group of specific compounds and adopting an integrated approach that incorporates VOs and/or broader phytochemical diversity. In metaphorical terms, thus far we have been observing this field through a window, as we have not yet been allowed to step through the door (Or we don't want to) and explore the external environment in its entirety and in an integrated manner.

## Conclusion

Despite the growing medicinal and industrial relevance of *Cannabis sativa* L., research on its VOs remains comparatively limited and methodologically heterogeneous, with few studies systematically evaluating how VO chemical composition responds to biotic and abiotic drivers. Further investigation is required to elucidate the determinants of volatilome variability and to support the optimization of cultivation and processing strategies aimed at producing high-quality, chemically consistent VOs. A particularly critical and still underexplored dimension is the spatial structuring of chemical variability across *Cannabis* germplasm and production territories, since geographically explicit chemotype patterns can clarify the relative contribution of genotype-by-environment interactions to chemical expression and, consequently, improve reproducibility, labeling accuracy, and product standardization.

At the global scale, this synthesis delineates three major terpene-based chemotypic domains organizing VO chemodiversity, consistent with a structured chemical landscape defined by monoterpene dominance, sesquiterpene-hydrocarbon scaffolds, and oxygenation-driven divergence. Importantly, these chemotypes are not static entities, but may undergo context-dependent shifts driven by phenotypic plasticity, environmental gradients, and post-harvest management. This is exemplified by Italy, where territoriality appears to generate region-specific “geotypes”, producing a mosaic of volatile profiles structured by elevation/latitude and coordinated chemical modules.

Chemotype- and chemodiversity-centered approaches can therefore provide an operational basis for improving the quality of *Cannabis* products; however, they should be coupled with longitudinal chemical stability assessments across developmental stages and along the full production pipeline. In this context, integrating agronomic descriptors (pre- and post-harvest parameters) with chemical and phenological tracking (crop chemical phenology, CCP) represents a robust starting point for advancing standardization under a “crop-to-shop” framework, in which cultivation decisions, processing conditions, packaging, and storage are treated as interconnected determinants of chemical integrity and final product performance.

## Supplementary Information

Supplementary Material 1.

Supplementary Material 2.

Supplementary Material 3.

Supplementary Material 4.

Supplementary Material 5.

Supplementary Material 6.

## Data Availability

All data generated or analyzed during this study are included in this published article [and its supplementary information files].

## Abbreviations

Δ^9^-THC: Δ^9^-Tetrahydrocarboncannabinol
CBD: Cannabidiol
CBG: Cannabigerol
THCV: Tetrahydrocannabivarin
CBDV: Cannabidivarin
CBC: Cannabichromene
PCA: Principal Component Analysis
HCA: Hierarchical Cluster Analysis
CBN: Cannabinol
