# Supplementary figures and images for: Cannabis: from crop to shop—some insights about stability to access quality control
Source: J Cannabis Res. 2026 Feb 23;8:45. doi: 10.1186/s42238-026-00409-9 (PMC13032246; doi:10.1186/s42238-026-00409-9)

Supplementary Figure SS1 PRISMA flow diagram illustrating the study selection process


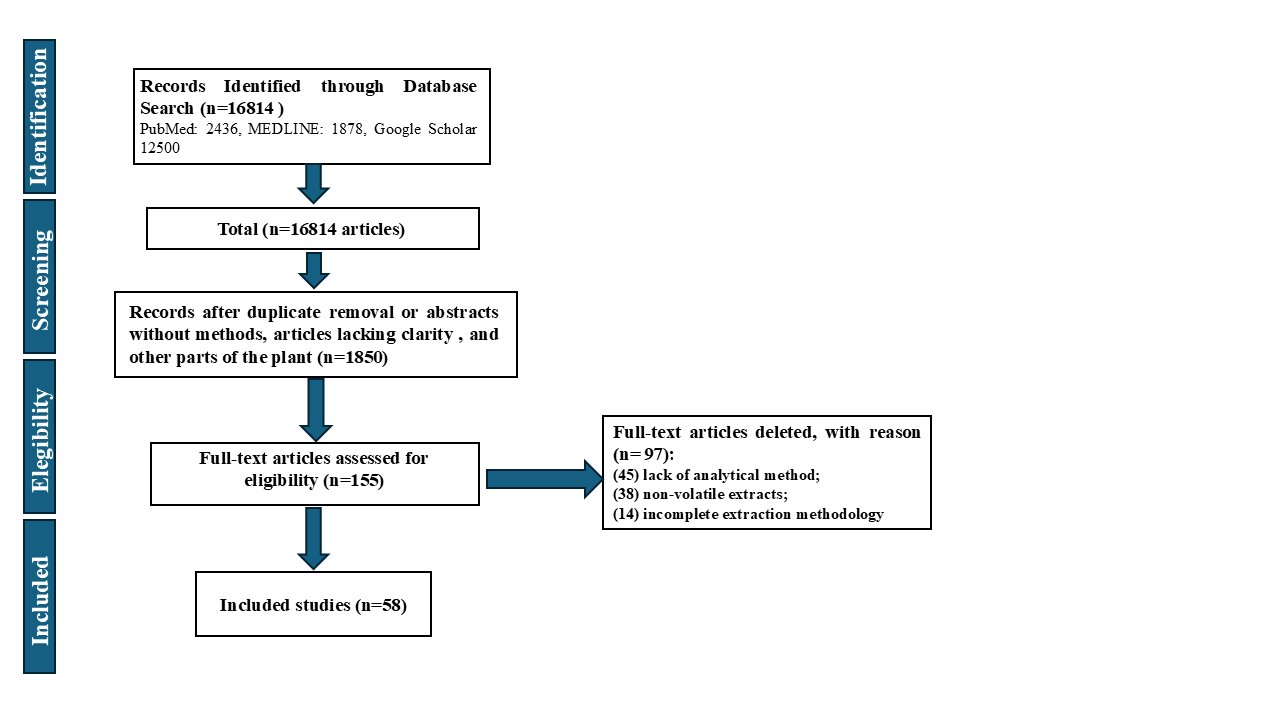

Supplement: Supplementary file 1 — Supplementary Material 1. [file 42238_2026_409_MOESM1_ESM.docx]
