# Supplementary material for: Cannabis: from crop to shop—some insights about stability to access quality control
Source: J Cannabis Res. 2026 Feb 23;8:45. doi: 10.1186/s42238-026-00409-9 (PMC13032246; doi:10.1186/s42238-026-00409-9)
Supplement: Supplementary file 2 — Supplementary Material 2. [file 42238_2026_409_MOESM2_ESM.docx]

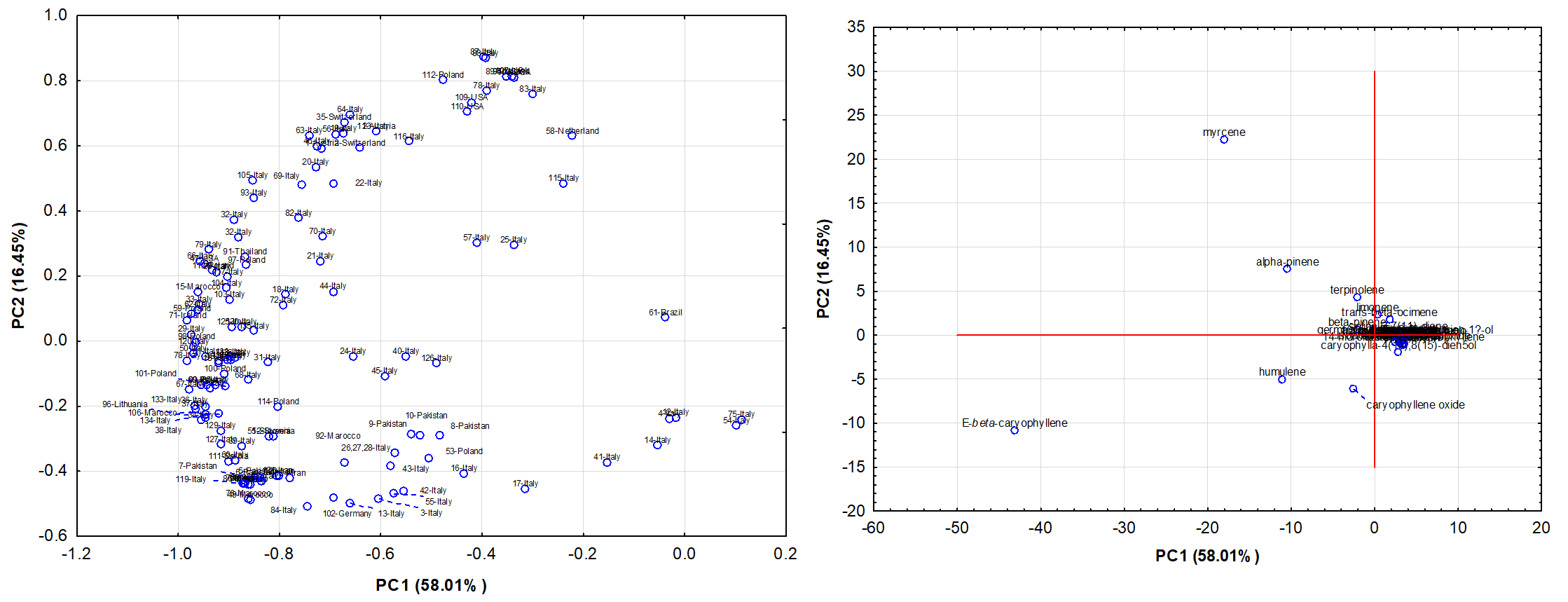


Supplementary Figure SS2. Principal component analysis (PCA) conducted on the complete composition of volatile oils extracted from all the *Cannabis s*pp. samples on a global scale
