# Supplementary material for: Cannabis: from crop to shop—some insights about stability to access quality control
Source: J Cannabis Res. 2026 Feb 23;8:45. doi: 10.1186/s42238-026-00409-9 (PMC13032246; doi:10.1186/s42238-026-00409-9)
Supplement: Supplementary file 3 — Supplementary Material 3. [file 42238_2026_409_MOESM3_ESM.docx]

Supplementary Table SS1 Majority contents (> 5% ) from Italy and Geographical distribution of *C. sativa* chemotypes

Legend GV= Genotype_Variety; CE= cinetic extraction; dry method 1= 1 week,1-3 mouth; min = minuts; dry method 2= 1 week dry + 1 mouth storage; dry method 3= 1 week dry + 3 mouth storage; Legends: MAE (Microwave-Assisted Extraction), HD (Hydrodistillation), SD (Steam Distillation), SCF (Supercritical Fluid Extraction), and ni (Not Identified)
