# Supplementary material for: Cannabis: from crop to shop—some insights about stability to access quality control
Source: J Cannabis Res. 2026 Feb 23;8:45. doi: 10.1186/s42238-026-00409-9 (PMC13032246; doi:10.1186/s42238-026-00409-9)
Supplement: Supplementary file 4 — Supplementary Material 4. [file 42238_2026_409_MOESM4_ESM.docx]

Supplementary Table SS1 Summary of Extraction Methods, and Literature References Codes from from Global (Figure 2) and Italian (Figure 5) Volatile Oil Studies

| **Code (Figure 2,5)** | **References** | **City** | **Country** | **Extraction** |
| --- | --- | --- | --- | --- |
| 1 | (116) | Amstetten | Austria | ni |
| 2 | (118) | Tenniken | Switzerland | HD |
| 3 | (25) | Rovigo | Italy | HD |
| 4 | (25) | Rovigo | Italy | HD |
| 5 | (20) | Kashimira | Pakistan | HD |
| 6 | (20) | Kashimira | Pakistan | SD |
| 7 | (20) | Kashimira | Pakistan | SCF |
| 8 | (20) | Kashimira | Pakistan | SD |
| 9 | (20) | Kashimira | Pakistan | HD |
| 10 | (20) | Kashimira | Pakistan | SCF |
| 11 | (42) | Burgenland | Austria | HD |
| 12 | (25) | Rovigo | Italy | HD |
| 13 | (25) | Rovigo | Italy | HD |
| 14 | (25) | Rovigo | Italy | HD |
| 15 | (25) | El Aiún | Marocco | HD |
| 16 | (25) | Rovigo | Italy | HD |
| 17 | (25) | Rovigo | Italy | HD |
| 18 | (31) | Ascoli Piceno | Italy | ni |
| 19 | (31) | Ascoli Piceno | Italy | ni |
| 20 | (31) | Ascoli Piceno | Italy | ni |
| 21 | (31) | Ascoli Piceno | Italy | ni |
| 22 | (31) | Ascoli Piceno | Italy | ni |
| 23 | (31) | Ascoli Piceno | Italy | ni |
| 24 | (31) | Ascoli Piceno | Italy | ni |
| 25 | (31) | Ascoli Piceno | Italy | ni |
| 29 | (23) | Fiuminata | Italy | HD |
| 30 | (23) | Fiuminata | Italy | SD |
| 31 | (23) | Fiuminata | Italy | HD |
| 32 | (68) | Fiuminata | Italy | MAE |
| 32 | (31) | Fiuminata | Italy | MAE |
| 33 | (29) | Fiuminata | Italy | HD |
| 34 | (72) | Ancona | Italy | MAE |
| 35 | (72,117) | Zurich | Switzerland | SD |
| 36 | (72) | Ancona | Italy | HD |
| 37 | (72) | San Severino, Marche | Italy | HD |
| 38 | (72) | San Severino, Marche | Italy | MAE |
| 39 | (89) | Santa Luce | Italy | HD |
| 40 | (40) | Foggia | Italy | HD |
| 41 | (25) | Rovigo | Italy | HD |
| 42 | (25) | Rovigo | Italy | HD |
| 43 | (26) | Rovigo | Italy | HD |
| 44 | (40) | Lucca | Italy | MAE |
| 45 | (40) | Lucca | Italy | HD |
| 46 | (67) | Po Valley | Italy | SD |
| 47 | (38) | San Clemente | USA | HD |
| 48 | (45) | Abruzzo | Italy | SD |
| 49 | (33) | Rif | Marocco | SD |
| 50 | (34) | Fiumata | Italy | HD, SD |
| 51 | (82) | Eslovênia | Slovenia | HD |
| 52 | (82) | Eslovênia | Slovenia | HD |
| 53 | (21) | - | Italy | SD |
| 54 | (25) | Rovigo | Italy | HD |
| 55 | (25) | Rovigo | Italy | HD |
| 56 | (69) | Amandola | Italy | HD |
| 57 | (69) | Amandola | Italy | HD |
| 58 | (169) | Netherland | Netherland | AV |
| 59 | (21) | Poznań | Polish | SD |
| 60 | (70) | Pisa Province | Italy | HD |
| 60 | (70) | Pistoia Province | Italy | HD |
| 61 | (27) | - | Brazil | ni |
| 62 | (35) | Fiuminata | Italy | MAE |
| 63 | (35) | Fiuminata | Italy |  |
| 64 | (119) | Eslovenia | Italy | SD |
| 65 | (74) | Perugia | Italy | HD |
| 66 | (19) | Friuli,Venezia | Italy | HD |
| 67 | (19) | Friuli,Venezia | Italy | SCF |
| 68 | (69) | Amandola | Italy | HD |
| 69 | (73) | Ragusa | Italy | HD-MAE |
| 70 | (120) | Pavia | Italy | HD-MAE |
| 71 | (122) | Dublin | Ireland | ni |
| 72 | (120) | Pavia | Italy | HD |
| 73 | (32) | Backi Petrovac | Serbia | HD |
| 74 | (32) | Kovacica | Serbia | HD |
| 75 | (25) | Rovigo | Italy | HD |
| 76 | (36) | northern Morocco | Marocco | SD |
| 77 | (29) | Fiuminata | Italy | SD |
| 78 | (76) | Tortoreto | Italy | SD |
| 78 | (76) | Tortoreto | Italy | SD |
| 79 | (19) | Friuli,Venezia | Italy | FSC |
| 80 | (22) | Chieti | Italy | HD |
| 81 | (30) | Udine | Italy | HD |
| 82 | (18) | Pisa | Italy | HD |
| 83 | (18) | Pisa | Italy | SPME |
| 84 | (70) | Pisa Province | Italy | HD |
| 86 | (178) | Taounate | Marocco | HD |
| 87 | (41) | Sassari, Region of Sardinia | Italy | HD |
| 88 | (41) | Sassari, Region of Sardinia | Italy | HD |
| 26,27,28 | (31) | Ascoli Piceno | Italy | MAE |
| 89 | (179) | Chiang Mai | Thailand | MAE |
| 89 | (179) | Chiang Mai | Thailand | MAE |
| 91 | (179) | Mukdahan | Thailand | MAE |
| 92 | (178) | Tafrant region, Taounate | Marocco | HD; SD; MAH |
| 93 | (180) | Carmagnola | Italy | HD; SD; MAH |
| 94 | (181) | Watkinsville, Georgia | USA | HD |
| 95 | (181) | Watkinsville, Georgia | USA | HD |
| 96 | (24) | Pavilnys, Vilnius | Lithuania | HD |
| 97 | (182) | Oborniki Śląskie | Poland | HD |
| 98 | (182) | Oborniki Śląskie | Poland | HD |
| 99 | (182) | Oborniki Śląskie | Poland | HD |
| 100 | (182) | Oborniki Śląskie | Poland | HD |
| 101 | (182) | Oborniki Śląskie | Poland | HD |
| 102 | (28) | Malsch | Germany | HD |
| 103 | (75) | Abruzzo | Italy | HD |
| 104 | (75) | Abruzzo | Italy | HD |
| 105 | (75) | Abruzzo | Italy | HD |
| 106 | (49) | Al-Hoceima province | Marocco | HD |
| 107 | (17) | Mississippi | USA | SD |
| 108 | (17) | Mississippi | USA | SD |
| 109 | (17) | Mississippi | USA | SD |
| 110 | (17) | Mississippi | USA | SD |
| 111 | (37) | Bački Petrovac | Serbia | SD |
| 112 | (37) | Pętkowo | Poland | SD |
| 113 | (183) | Wrocław | Poland | SD |
| 114 | (183) | Wrocław | Poland | SD |
| 115 | (46) | Naples | Italy | HD |
| 116 | (32) | Abruzzo | Italy | HD |
| 117 | (32) | Abruzzo | Italy | HD |
| 118 | (32) | Abruzzo | Italy | HD |
| 119 | (32) | Abruzzo | Italy | HD |
| 120 | (32) | Abruzzo | Italy | HD |
| 121 | (48) | Karaj | Iran | HD |
| 122 | (48) | Karaj | Iran | HD |
| 123 | (48) | Karaj | Iran | HD |
| 124 | (48) | Karaj | Iran | HD |
| 125 | (71) | Fiuminata | Italy | HD |
| 126 | (71) | Fiuminata | Italy | HD |
| 127 | (71) | Fiuminata | Italy | HD |
| 128 | (71) | Fiuminata | Italy | HD |
| 129 | (71) | Fiuminata | Italy | HD |
| 130 | (71) | Fiuminata | Italy | SD |
| 131 | (71) | Fiuminata | Italy | SD |
| 132 | (71) | Fiuminata | Italy | SD |
| 133 | (71) | Fiuminata | Italy | SD |
| 134 | (71) | Fiuminata | Italy | SD |
| 135 | (71) | Fiuminata | Italy | MAH |
| 136 | (71) | Fiuminata | Italy | MAH |
| 137 | (71) | Fiuminata | Italy | MAH |
| 138 | (71) | Fiuminata | Italy | MAH |
| 139 | (71) | Fiuminata | Italy | MAH |

Legends: MAE (Microwave-Assisted Extraction), HD (Hydrodistillation), SD (Steam Distillation), SCF (Supercritical Fluid Extraction), and ni (Not Identified).
