# Supplementary material for: Cannabis: from crop to shop—some insights about stability to access quality control
Source: J Cannabis Res. 2026 Feb 23;8:45. doi: 10.1186/s42238-026-00409-9 (PMC13032246; doi:10.1186/s42238-026-00409-9)
Supplement: Supplementary file 5 — Supplementary Material 5. [file 42238_2026_409_MOESM5_ESM.docx]

Supplementary Table SS3 Global PCA loading matrix.

|  | **GLOBAL PCA** | | | | | |
| --- | --- | --- | --- | --- | --- | --- |
|  | **PC1** | **PC2** |  |  | **PC1** | **PC2** |
| alpha-pinene | -17.9936 | 22.2185 |  | 106-Marocco | -0.918699 | -0.224358 |
| beta-pinene | 1.8374 | 1.7416 |  | 115-Italy | -0.237572 | 0.483588 |
| myrcene | 0.3718 | 2.2730 |  | 58-Netherland | -0.221490 | 0.628864 |
| *trans*-beta-ocimene | -2.0605 | 4.2237 |  | 82-Italy | -0.760890 | 0.377329 |
| limonene | 3.0609 | -0.8845 |  | 83-Italy | -0.298505 | 0.757373 |
| terpinolene | 2.9825 | -0.8293 |  | 54-Italy | 0.103858 | -0.260666 |
| eucaliptol | 3.5621 | -0.2237 |  | 41-Italy | -0.151853 | -0.373902 |
| linalool | 3.5263 | -0.4601 |  | 12-Italy | -0.016783 | -0.235786 |
| terpineol | 3.5057 | -0.4381 |  | 13-Italy | -0.660161 | -0.497535 |
| linalyl acetate | 3.2287 | -0.7875 |  | 14-Italy | -0.052257 | -0.322348 |
| estragole | 3.6208 | -0.3164 |  | 75-Italy | 0.113436 | -0.242472 |
| menthol | 3.2835 | -0.6459 |  | 55-Italy | -0.573404 | -0.469452 |
| carvone | 3.5542 | -0.3795 |  | 3-Italy | -0.602834 | -0.484075 |
| *Z*-caryophyllene | 3.6556 | -0.1952 |  | 4-Italy | -0.029574 | -0.238657 |
| *trans*-alpha-bergamoteno | 3.3639 | -0.5510 |  | 16-Italy | -0.434312 | -0.407099 |
| gamma-elemene | -43.0810 | -10.8249 |  | 17-Italy | -0.313288 | -0.454995 |
| *cis*-beta-farnesene | -11.0628 | -5.0519 |  | 42-Italy | -0.553654 | -0.463577 |
| E-beta-caryophyllene | 3.5995 | -0.2421 |  | 43-Italy | -0.579378 | -0.386255 |
| humulene | 2.8670 | 0.1339 |  | 44-Italy | -0.693388 | 0.151423 |
| beta-selinene | 3.5913 | -0.2745 |  | 45-Italy | -0.591433 | -0.108159 |
| selina-3,7(11)-diene | 3.3419 | -0.4859 |  | 46-Italy | -0.724864 | 0.597428 |
| delta-cadinene | -2.5093 | -6.1627 |  | 47-USA | -0.947726 | 0.236092 |
| nerolidol | 3.5052 | -0.9401 |  | 70-Italy | -0.713946 | 0.319996 |
| caryophyllene oxide | 3.6148 | -0.8898 |  | 72-Italy | -0.791370 | 0.109334 |
| isoaromadendrene epoxide | 3.4922 | -1.0504 |  | 73-Serbia | -0.869692 | -0.438267 |
| humulene oxyde II | 3.5059 | -1.0324 |  | 111-Serbia | -0.900460 | -0.370591 |
| beta-eudesmol | 3.3249 | -1.1805 |  | 74-Serbia | -0.861297 | -0.441138 |
| beta-bisabolene | 3.1774 | -0.3119 |  | 107-USA | -0.340550 | 0.813459 |
| cubenol | 3.6735 | -0.2613 |  | 108-USA | -0.336461 | 0.808435 |
| guaiol | 3.1372 | -0.3158 |  | 109-USA | -0.420114 | 0.731260 |
| germacra-4(15),5,10(14)-trien-1-ol | 2.8824 | -1.9998 |  | 110-USA | -0.428292 | 0.703269 |
| 10-epi-gamma-eudesmol | 3.4619 | -1.1029 |  | 80-Italy | -0.925819 | -0.136210 |
| caryophylla-4(14),8(15)-dien5ol | 2.4489 | -0.8455 |  | 81-Italy | -0.944155 | -0.047972 |
| 14-hidroxi-9 epi(E) caryophyllene |  |  |  | 11-Austria | -0.608853 | 0.643260 |
| alpha-bisabolol |  |  |  | 113-Poland | -0.931142 | 0.215494 |
|  |  |  |  | 114-Poland | -0.802810 | -0.202745 |
|  |  |  |  | 96-Lithuania | -0.944895 | -0.237290 |
|  |  |  |  | 91-Thailand | -0.867752 | 0.258155 |
|  |  |  |  | 112-Poland | -0.477266 | 0.800606 |
|  |  |  |  | 18-Italy | -0.786703 | 0.142451 |
|  |  |  |  | 19-Italy | -0.672776 | 0.636272 |
|  |  |  |  | 20-Italy | -0.727597 | 0.534423 |
|  |  |  |  | 21-Italy | -0.718107 | 0.244182 |
|  |  |  |  | 22-Italy | -0.691682 | 0.482691 |
|  |  |  |  | 23-Italy | -0.608581 | 0.643902 |
|  |  |  |  | 24-Italy | -0.653673 | -0.047352 |
|  |  |  |  | 25-Italy | -0.336836 | 0.293759 |
|  |  |  |  | 26,27,28-Italy | -0.572075 | -0.344333 |
|  |  |  |  | 71-Ireland | -0.981083 | 0.061372 |
|  |  |  |  | 59-Poland | -0.972479 | 0.083162 |
|  |  |  |  | 102-Germany | -0.692692 | -0.482873 |
|  |  |  |  | 34-Italy | -0.953958 | -0.244041 |
|  |  |  |  | 36-Italy | -0.967395 | -0.199320 |
|  |  |  |  | 37-Italy | -0.965577 | -0.209115 |
|  |  |  |  | 38-Italy | -0.953958 | -0.244041 |
|  |  |  |  | 39-Italy | -0.872934 | -0.323713 |
|  |  |  |  | 40-Italy | -0.548883 | -0.047196 |
|  |  |  |  | 94-USA | -0.349930 | 0.811388 |
|  |  |  |  | 95-USA | -0.349930 | 0.811388 |
|  |  |  |  | 87-Italy | -0.396250 | 0.870767 |
|  |  |  |  | 88-Italy | -0.392189 | 0.867455 |
|  |  |  |  | 77-Italy | -0.922699 | 0.211613 |
|  |  |  |  | 93-Italy | -0.849250 | 0.439447 |
|  |  |  |  | 78-Italy | -0.980914 | -0.060739 |
|  |  |  |  | 78-Italy | -0.389654 | 0.768691 |
|  |  |  |  | 51-Slovenia | -0.819630 | -0.293906 |
|  |  |  |  | 52-Slovenia | -0.810774 | -0.294096 |
|  |  |  |  | 84-Italy | -0.745225 | -0.509049 |
|  |  |  |  | 60-Italy | -0.953107 | -0.136851 |
|  |  |  |  | 60-Italy | -0.887274 | -0.366989 |
|  |  |  |  | 1-Austria | -0.715381 | 0.591582 |
|  |  |  |  | 48-Italy | -0.918329 | -0.062111 |
|  |  |  |  | 89-Thailand | -0.349930 | 0.811388 |
|  |  |  |  | 50-Italy | -0.969762 | -0.038801 |
|  |  |  |  | 32-Italy | -0.888588 | 0.372053 |
|  |  |  |  | 35-Switzerland | -0.671513 | 0.670271 |
|  |  |  |  | 29-Italy | -0.973087 | 0.020453 |
|  |  |  |  | 30-Italy | -0.922699 | 0.211613 |
|  |  |  |  | 31-Italy | -0.823015 | -0.066224 |
|  |  |  |  | 32-Italy | -0.880142 | 0.317644 |
|  |  |  |  | 33-Italy | -0.957746 | 0.112215 |
|  |  |  |  | 62-Italy | -0.960245 | 0.096224 |
|  |  |  |  | 63-Italy | -0.740035 | 0.629564 |
|  |  |  |  | 125-Italy | -0.892922 | 0.043404 |
|  |  |  |  | 126-Italy | -0.488792 | -0.069098 |
|  |  |  |  | 127-Italy | -0.915008 | -0.318621 |
|  |  |  |  | 128-Italy | -0.857284 | -0.441321 |
|  |  |  |  | 129-Italy | -0.915795 | -0.275322 |
|  |  |  |  | 130-Italy | -0.874774 | 0.042652 |
|  |  |  |  | 131-Italy | -0.919593 | -0.070164 |
|  |  |  |  | 132-Italy | -0.937034 | -0.144626 |
|  |  |  |  | 133-Italy | -0.945187 | -0.202114 |
|  |  |  |  | 134-Italy | -0.944716 | -0.227037 |
|  |  |  |  | 135-Italy | -0.850184 | 0.030710 |
|  |  |  |  | 136-Italy | -0.887355 | -0.051089 |
|  |  |  |  | 137-Italy | -0.902752 | -0.060036 |
|  |  |  |  | 138-Italy | -0.894948 | -0.049968 |
|  |  |  |  | 139-Italy | -0.894879 | -0.059887 |
|  |  |  |  | 64-Italy | -0.659258 | 0.695991 |
|  |  |  |  | 65-Italy | -0.870851 | -0.436770 |
|  |  |  |  | 49-Marocco | -0.857444 | -0.488847 |
|  |  |  |  | 56-Italy | -0.688019 | 0.633900 |
|  |  |  |  | 57-Italy | -0.409762 | 0.300730 |
|  |  |  |  | 68-Italy | -0.861306 | -0.120325 |
|  |  |  |  | 69-Italy | -0.754866 | 0.477463 |
|  |  |  |  | 86-Marocco | -0.866616 | -0.440044 |
|  |  |  |  | 15-Marocco | -0.960824 | 0.150265 |
|  |  |  |  | 2-Switzerland | -0.640067 | 0.591888 |
|  |  |  |  | 66-Italy | -0.954785 | 0.244174 |
|  |  |  |  | 67-Italy | -0.977393 | -0.148758 |
|  |  |  |  | 79-Italy | -0.939469 | 0.280390 |
|  |  |  |  | 121-Iran | -0.803799 | -0.413708 |
|  |  |  |  | 122-Iran | -0.834810 | -0.431684 |
|  |  |  |  | 123-Iran | -0.800252 | -0.416391 |
|  |  |  |  | 124-Iran | -0.778585 | -0.423078 |
|  |  |  |  | 5-Pakistan | -0.842101 | -0.416757 |
|  |  |  |  | 6-Pakistan | -0.846994 | -0.421109 |
|  |  |  |  | 7-Pakistan | -0.836992 | -0.421914 |
|  |  |  |  | 8-Pakistan | -0.482973 | -0.290634 |
|  |  |  |  | 9-Pakistan | -0.522118 | -0.291028 |
|  |  |  |  | 10-Pakistan | -0.539085 | -0.286472 |
|  |  |  |  | 53-Poland | -0.504113 | -0.362792 |
|  |  |  |  | 61-Brazil | -0.036723 | 0.071286 |
|  |  |  |  | 103-Italy | -0.897934 | 0.127471 |
|  |  |  |  | 104-Italy | -0.904046 | 0.162120 |
|  |  |  |  | 105-Italy | -0.852082 | 0.491761 |
|  |  |  |  | 116-Italy | -0.543587 | 0.612066 |
|  |  |  |  | 117-Italy | -0.901340 | 0.196017 |
|  |  |  |  | 118-Italy | -0.870051 | -0.437906 |
|  |  |  |  | 119-Italy | -0.870043 | -0.437915 |
|  |  |  |  | 120-Italy | -0.968336 | -0.018628 |
|  |  |  |  | 92-Marocco | -0.671646 | -0.375167 |
|  |  |  |  | 76-Marocco | -0.861668 | -0.485593 |
|  |  |  |  | 97-Poland | -0.865121 | 0.232440 |
|  |  |  |  | 98-Poland | -0.963587 | -0.003089 |
|  |  |  |  | 99-Poland | -0.942472 | -0.136916 |
|  |  |  |  | 100-Poland | -0.907270 | -0.101312 |
|  |  |  |  | 101-Poland | -0.906583 | -0.139521 |
