# Supplementary material for: Cannabis: from crop to shop—some insights about stability to access quality control
Source: J Cannabis Res. 2026 Feb 23;8:45. doi: 10.1186/s42238-026-00409-9 (PMC13032246; doi:10.1186/s42238-026-00409-9)
Supplement: Supplementary file 6 — Supplementary Material 6. [file 42238_2026_409_MOESM6_ESM.docx]

Supplementary Table SS4 Italy PCA loading matrix.

| **ITALY - PCA** | | | | | | |  |
| --- | --- | --- | --- | --- | --- | --- | --- |
|  |  |  |  |  |  |  |  |
|  | **PC1** | **PC2** |  |  | **PC1** | **PC2** |  |
| α-pinene | -0.15035 | 0.25822 |  | 115-Naples | -0.35417 | 1.77766 |  |
| β-pinene | -0.08243 | 0.0135 |  | 82-Pisa | -0.35264 | 0.96793 |  |
| myrcene | -0.14309 | 0.32058 |  | 83-Pisa | 0.06058 | 1.57236 |  |
| *trans*-β-ocimene | -0.03893 | 0.18228 |  | 54-Rovigo | 4.04294 | 0.38159 |  |
| limonene | -0.04184 | 0.19601 |  | 41-Rovigo | 3.53635 | 0.30968 |  |
| terpinolene | -0.06206 | 0.14964 |  | 12-Rovigo | 0.76861 | -1.17447 |  |
| terpineol | -0.017 | -0.00993 |  | 13-Rovigo | 0.49769 | -1.39639 |  |
| *E*-β-caryophyllene | -0.14877 | -0.36393 |  | 14-Rovigo | 4.21182 | 0.50946 |  |
| α-humulene | -0.1032 | -0.44195 |  | 75-Rovigo | 4.22414 | 0.67167 |  |
| β-selinene | -0.01477 | 0.03979 |  | 55-Rovigo | 0.20566 | -1.33082 |  |
| selina-3,7(11)-diene | -0.03176 | 0.29819 |  | 3-Rovigo | 0.1952 | -1.40751 |  |
| δ-cadinene | -0.01652 | -0.11997 |  | 4-Rovigo | 0.69849 | -1.0952 |  |
| caryophyllene oxide | 0.18715 | -0.24484 |  | 16-Rovigo | 0.73191 | -1.14433 |  |
| isoaromadendrene epoxide | 0.37374 | 0.06064 |  | 17-Rovigo | 3.58688 | 0.07915 |  |
| humulene oxyde II | 0.37374 | 0.06064 |  | 42-Rovigo | 0.92898 | -0.89708 |  |
| β-eudesmol | 0.37374 | 0.06064 |  | 43-Rovigo | -0.04312 | -0.0646 |  |
| β-bisabolene | 0.37374 | 0.06064 |  | 44-Lucca | -0.29517 | 0.54219 |  |
| cubenol | 0.37374 | 0.06064 |  | 45-Lucca | -0.30577 | 0.36434 |  |
| guaiol | -0.02828 | 0.29315 |  | 46-Po Valley | -0.36021 | 0.80821 |  |
| 10-epi-γ-eudesmol | -0.02837 | 0.29616 |  | 70-Pavia | -0.31196 | 0.8967 |  |
| caryophylla-4(14),8(15)-dien5ol | 0.18292 | -0.18293 |  | 72-Pavia | -0.34868 | 0.66371 |  |
| 14-hidroxi-9 epi(E) caryophyllene | 0.27875 | -7.99E-04 |  | 80-Chieti | -0.1819 | -0.5854 |  |
| α-bisabolol | -0.01682 | 0.12685 |  | 81-Udine | -0.36498 | -0.26758 |  |
| 14-hidroxi9 epi(E) caryophyllene | 0.24876 | 0.01298 |  | 18-Ascoli Piceno | -0.34245 | 1.73179 |  |
| α-bisabolol | 0.00761 | 0.04322 |  | 19-Ascoli Piceno | -0.30696 | 1.11867 |  |
|  |  |  |  | 20-Ascoli Piceno | -0.32313 | 1.24034 |  |
|  |  |  |  | 21-Ascoli Piceno | -0.39149 | 1.80482 |  |
|  |  |  |  | 22-Ascoli Piceno | -0.33581 | 1.48078 |  |
|  |  |  |  | 23-Ascoli Piceno | -0.39043 | 0.95613 |  |
|  |  |  |  | 24-Ascoli Piceno | -0.31399 | 2.61702 |  |
|  |  |  |  | 25-Ascoli Piceno | -0.24647 | 1.3973 |  |
|  |  |  |  | 26,27,28-Ascoli Piceno | -0.31324 | 2.52602 |  |
|  |  |  |  | 34-Ancona | -0.24678 | -0.83296 |  |
|  |  |  |  | 36-Ancona | -0.25846 | -0.78518 |  |
|  |  |  |  | 37-San Severino, Marche | -0.2579 | -0.79867 |  |
|  |  |  |  | 38-San Severino, Marche | -0.24678 | -0.83296 |  |
|  |  |  |  | 39-Santa Luce | -0.24468 | -0.73831 |  |
|  |  |  |  | 40-Foggia | -0.3938 | -1.82668 |  |
|  |  |  |  | 87-Sassari, region of Sardinia | -0.36277 | 1.43101 |  |
|  |  |  |  | 88-Sassari, region of Sardinia | -0.36479 | 1.42285 |  |
|  |  |  |  | 77-Fiuminata | -0.3577 | 0.27495 |  |
|  |  |  |  | 93-Carmagnola | -0.33481 | 0.29039 |  |
|  |  |  |  | 78-Tortoreto | -0.35482 | -0.55139 |  |
|  |  |  |  | 78-Tortoreto | -0.40942 | 1.64884 |  |
|  |  |  |  | 84-Pisa Province | 0.12673 | -1.4518 |  |
|  |  |  |  | 60-Pisa Province | -0.34199 | -0.54255 |  |
|  |  |  |  | 60-Pistoia Province | -0.23981 | -0.90499 |  |
|  |  |  |  | 48-Abruzzo | -0.37574 | -0.29414 |  |
|  |  |  |  | 50-Fiuminata | -0.35534 | -0.23477 |  |
|  |  |  |  | 32-Fiuminata | -0.36372 | 0.30632 |  |
|  |  |  |  | 29-Fiuminata | -0.34085 | -0.27676 |  |
|  |  |  |  | 30-Fiuminata | -0.3577 | 0.27495 |  |
|  |  |  |  | 31-Fiuminata | -0.12021 | -0.40919 |  |
|  |  |  |  | 32-Fiuminata | -0.36513 | 0.41813 |  |
|  |  |  |  | 33-Fiuminata | -0.34081 | 0.06847 |  |
|  |  |  |  | 62-Fiuminata | -0.27094 | 0.0503 |  |
|  |  |  |  | 63-Fiuminata | -0.36062 | 0.58739 |  |
|  |  |  |  | 125-Fiuminata | -0.29437 | -0.27809 |  |
|  |  |  |  | 126-Fiuminata | -- | -- |  |
|  |  |  |  | 127-Fiuminata | -0.2452 | -1.26642 |  |
|  |  |  |  | 128-Fiuminata | -0.33684 | -1.69861 |  |
|  |  |  |  | 129-Fiuminata | -0.33884 | -1.29476 |  |
|  |  |  |  | 130-Fiuminata | -0.30547 | -0.28015 |  |
|  |  |  |  | 131-Fiuminata | -0.2978 | -0.45622 |  |
|  |  |  |  | 132-Fiuminata | -0.28382 | -0.5959 |  |
|  |  |  |  | 133-Fiuminata | -0.28765 | -0.7316 |  |
|  |  |  |  | 134-Fiuminata | -0.29547 | -0.79953 |  |
|  |  |  |  | 135-Fiuminata | -0.32556 | -0.27575 |  |
|  |  |  |  | 136-Fiuminata | -0.30575 | -0.42252 |  |
|  |  |  |  | 137-Fiuminata | -0.30915 | -0.44374 |  |
|  |  |  |  | 138-Fiuminata | -0.32061 | -0.41639 |  |
|  |  |  |  | 139-Fiuminata | -0.32213 | -0.42423 |  |
|  |  |  |  | 64-Eslovenia | -0.36757 | 0.91498 |  |
|  |  |  |  | 65-Perugia | -0.3295 | -1.5712 |  |
|  |  |  |  | 56-Amandola | -0.3163 | 0.98671 |  |
|  |  |  |  | 57-Amandola | -0.25962 | 0.84843 |  |
|  |  |  |  | 68-Amandola | -0.15494 | -0.14513 |  |
|  |  |  |  | 69-Ragusa | -0.37567 | 0.7403 |  |
|  |  |  |  | 66-Friuli,Venezia | -0.34823 | 0.08087 |  |
|  |  |  |  | 67-Friuli,Venezia | -0.28726 | -0.49113 |  |
|  |  |  |  | 79-Friuli,Venezia | -0.34239 | 0.13855 |  |
|  |  |  |  | 103-Abruzzo | -0.3822 | -0.05879 |  |
|  |  |  |  | 104-Abruzzo | -0.40356 | -0.12687 |  |
|  |  |  |  | 105-Abruzzo | -0.35286 | 0.31834 |  |
|  |  |  |  | 116-Abruzzo | -0.40782 | 0.94451 |  |
|  |  |  |  | 117-Abruzzo | -0.38346 | -0.01258 |  |
|  |  |  |  | 118-Abruzzo | -0.33055 | -1.5894 |  |
|  |  |  |  | 119-Abruzzo | -0.33055 | -1.58951 |  |
|  |  |  |  | 120-Abruzzo | -0.37021 | -0.3309 |  |
